# Comparative Genomic Evidence for a Complete Nuclear Pore Complex in the Last Eukaryotic Common Ancestor

**DOI:** 10.1371/journal.pone.0013241

**Published:** 2010-10-08

**Authors:** Nadja Neumann, Daniel Lundin, Anthony M. Poole

**Affiliations:** 1 Department of Molecular Biology and Functional Genomics, Stockholm University, Stockholm, Sweden; 2 School of Biological Sciences and Biomolecular Interaction Centre, University of Canterbury, Christchurch, New Zealand; Institut de Genetique et Microbiologie, France

## Abstract

**Background:**

The Nuclear Pore Complex (NPC) facilitates molecular trafficking between nucleus and cytoplasm and is an integral feature of the eukaryote cell. It exhibits eight-fold rotational symmetry and is comprised of approximately 30 nucleoporins (Nups) in different stoichiometries. Nups are broadly conserved between yeast, vertebrates and plants, but few have been identified among other major eukaryotic groups.

**Methodology/Principal Findings:**

We screened for Nups across 60 eukaryote genomes and report that 19 Nups (spanning all major protein subcomplexes) are found in all eukaryote supergroups represented in our study (Opisthokonts, Amoebozoa, Viridiplantae, Chromalveolates and Excavates). Based on parsimony, between 23 and 26 of 31 Nups can be placed in LECA. Notably, they include central components of the anchoring system (Ndc1 and Gp210) indicating that the anchoring system did not evolve by convergence, as has previously been suggested. These results significantly extend earlier results and, importantly, unambiguously place a fully-fledged NPC in LECA. We also test the proposal that transmembrane Pom proteins in vertebrates and yeasts may account for their variant forms of mitosis (open mitoses in vertebrates, closed among yeasts). The distribution of homologues of vertebrate Pom121 and yeast Pom152 is not consistent with this suggestion, but the distribution of fungal Pom34 fits a scenario wherein it was integral to the evolution of closed mitosis in ascomycetes. We also report an updated screen for vesicle coating complexes, which share a common evolutionary origin with Nups, and can be traced back to LECA. Surprisingly, we find only three supergroup-level differences (one gain and two losses) between the constituents of COPI, COPII and Clathrin complexes.

**Conclusions/Significance:**

Our results indicate that all major protein subcomplexes in the Nuclear Pore Complex are traceable to the Last Eukaryotic Common Ancestor (LECA). In contrast to previous screens, we demonstrate that our conclusions hold regardless of the position of the root of the eukaryote tree.

## Introduction

**Figure 1:**
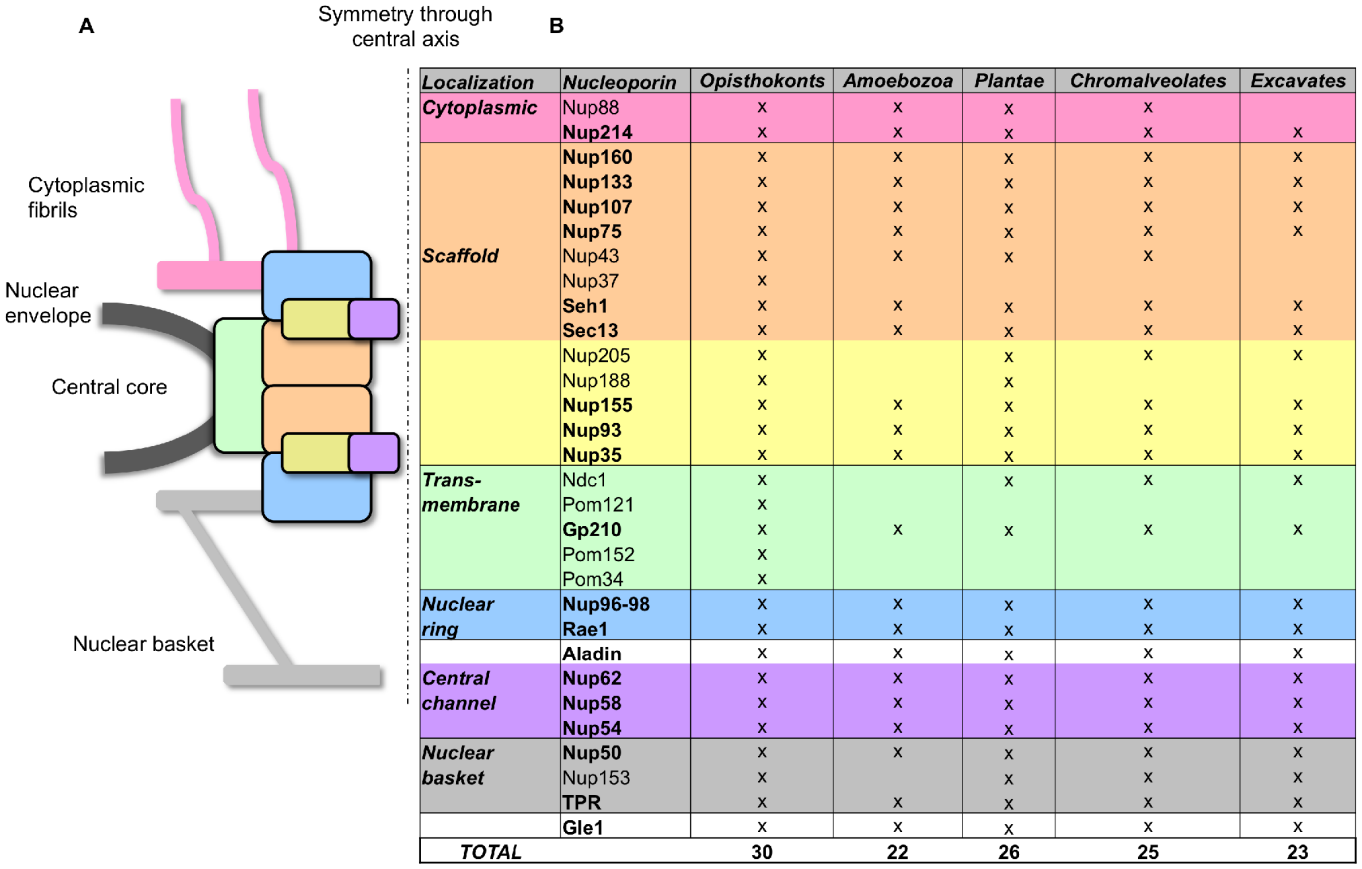
NPC structure, composition and Nup conservation across eukaryotes. a) Schematic section through the nuclear pore complex. Sub-complexes are indicated as boxes and marked in different colors to indicate their position in the pore. b) The table summarizes Nup distribution across eukaryotic super-groups. Color-coding matches that of the subcomplexes in (a). Nucleoporins indicated with bold letters are universally distributed across eukaryotes, as judged by presence in at least one genome from each of the five supergroups.

Nuclear pore complexes (NPCs) mediate molecular trafficking between nucleus and cytoplasm [1], [2]. They are composed of ∼30 different proteins, called nucleoporins (Nups), that are present in multiple copies in each pore [3], [4], [5]. Most Nups are constituents of specific sub-complexes, which form the major structural units of the pore: cytoplasmic fibrils, central core and the nuclear basket (Figure 1a) [6], [7].

The majority of Nups are conserved between mammals and yeasts [3], [4], [7], [8] and previous genomic studies demonstrate extensive conservation of the NPC also in plants and eukaryotic algae [9], [10], [11].

The extent of conservation of NPC components outside these groups appears patchy however [10], [11]. As Mans et al. [10] acknowledged, this makes it difficult to unambiguously establish the complexity of the NPC in the Last Eukaryotic Common Ancestor (LECA), since inferences are dependent upon the position of the root of the eukaryote tree. Bapteste et al. [11], reporting a comparable distribution of Nups to Mans et al., noted furthermore that proteins involved in anchoring the NPC to the nuclear envelope were limited in their distribution. On the basis of this observation, Bapteste et al. concluded that the NPC anchoring system appears to have evolved multiple times independently.

This conclusion is moreover interesting in light of the recent suggestion that the yeast-specific transmembrane Nups Pom152 and Pom34 may be intimately linked to the evolution of closed mitosis in yeast [12]. Closed mitosis is not restricted to yeasts, as it is also observed in a range of protists [13], [14]. This raises the question as to whether the evolutionary lability of the anchoring system broadly correlates with the evolution of closed mitosis.

In the wider context of eukaryote origins, there is great value in the identification of Nup homologues in either archaea or bacteria, since this may shed light on the evolutionary origins of the nucleus. If Nups display similarity to proteins from either or both of these domains, the role of these proteins may provide new insights into the evolutionary emergence of key protein families or folds [10]. In this respect there has also been considerable interest in the nuclear envelope-like internal membranes observed in planctomycete bacteria [15], [16], and whether the putative pores identified from morphological data are constructed from protein components with similarity to eukaryote Nups. To date, no homologs to Nups have been identified in the genome of any planctomycete.

An alternative hypothesis, in principle compatible with several theories on eukaryote origins, is that the nucleus evolved autogenously in the eukaryote stem lineage [17], [18]. The protocoatomer hypothesis [18] in particular addresses the evolution of the NPC in detail. In brief, this model posits that the NPC and vesicle-coating complexes evolved from a rudimentary membrane-bending apparatus that generated internal structure through invagination. Devos et al.[18] reported that an NPC subcomplex (yeast Nup84/vertebrate Nup107-160) bears a striking resemblance to vesicle-coating complexes, both containing proteins with a unique β-propeller/α-solenoid architecture. Moreover, Sec13 is a component of both the NPC and the COPII vesicle-coating complex [19], [20]. Mans et al. [10] also noted similarities between NPC and vesicle-coating complex components, coming to a similar conclusion on the basis of sequence analyses.

Rapid progress in eukaryote genome sequence projects provides an ideal opportunity to revisit these questions with the benefit of a more comprehensive dataset. We report here the results of a screen covering 60 eukaryote genomes (representing five supergroups) with the aim of examining the extent to which protein subcomplexes that comprise the NPC are conserved across eukaryotes. We have also examined whether coatomer proteins from the COPI, COPII and clathrin complexes are as broadly conserved as NPC complex proteins, since an early common origin for both the NPC and vesicle coating complexes predicts this. Our results provide further support for a complete NPC in LECA and, in contrast to earlier studies, we show that this conclusion holds regardless of the position of the eukaryote root. We conclude that at least 23 and possibly as many as 26 nucleoporins, including key components of the anchoring system, were already present in LECA. We also report that the distribution of Pom34, but not Pom152, correlates with the occurrence of closed mitosis among fungi. Despite extensive searches, our screen did not recover clear Nup homologs in either bacterial or archaeal genomes, consistent with the view that the nuclear pore complex evolved within the eukaryote stem, after the divergence of archaea and eukaryotes.

## Results and Discussion

### Establishing the accuracy of HMMer-based identification of Nucleoporins

*In silico* gene annotation by sequence similarity is expected to be subject to a significant degree of error (and perhaps subjectivity), and in the current case is also complicated by the great evolutionary distances spanning the eukaryote tree. The recent publication of nucleoporins identified in *Trypanosoma brucei* using experimental proteomics and structure prediction approaches [32], provided us with a fortuitous internal control by which to test the accuracy of our *in silico* screens for eukaryote Nup homologs. As we had already completed our screen of *T. brucei* when the DeGrasse et al. study [32] was published, we were able to use the identified Nups reported therein as a blind control, in the spirit of CASP and CAPRI community experiments to test *ab initio* 3D protein structure and protein-protein docking prediction methods (reviewed in [33], [34]). Comparison of the candidates identified using our HMMer-based approach with the results reported by DeGrasse et al. is particularly useful in that *T. brucei* is an outgroup to all sequences included in our training dataset. Table 1 compares our predictions with those experimentally shown to be NPC components in *T. brucei*. As is evident from Table 1, our predictions accurately identified homologs for 10/10 Nups where DeGrasse et al. concluded orthology could be established. In our analysis, we predicted a Seh1 candidate not identified by DeGrasse et al., which could potentially be a false positive identification. Careful examination of the full Seh1 alignment (see supplementary file SI4) reveals that all sequence identities are contained within the six WD-repeat regions (though we note considerable sequence complexity within these regions, and clear motifs for WD repeats 1 and 3). Seh1 and Sec13 sequences can be difficult to distinguish, though, for *T. brucei*, DeGrasse et al. and our analyses independently identified the same Sec13 candidate. While absence of Seh1 sequence identities outside of the WD-repeat regions warrants caution, we were unable to identify any other candidate sequences with this same repeat architecture, suggesting this sequence may well be a Seh1 candidate, albeit a weak one. It is also worth noting that Seh1 is known not to be strongly associated with the Nup107-160 complex, which may explain its absence from proteomics data. DeGrasse et al. also identified an additional 13 proteins, seven of which carry FG repeats. It is to be expected that comparative approaches will tend to underestimate the components of any given complex, since the approach is dependent upon the starting dataset. Moreover, as FG-repeat proteins often carry no other distinguishing features, we deemed the presence of FG-repeats alone insufficient for assigning membership to the NPC, and such candidates were excluded from our study (Table 1). From the perspective of the current study, the results indicate that the HMMer-based approach used here is conservative but accurate, as no incorrect assignments were made in our control screen of *T. brucei*.

**Table 1:** Comparison of performance of HMMer-based Nup screen on *Trypanosoma brucei* by reference to published experimental data [32].

Nucleoporins identified experimentally by DeGrasse et al. 2009	Nups also identified using HMMer (this study)	Annotation	Returned by HMMer but excluded^a^	E-value^b^	Notes
Tb10.61.2630	+	Sec13		4.4e-89	
−	Tb11.01.5410	Seh1		9.1e-07	
Tb11.02.2120	+	Aladin		1.1e-12	
Tb09.160.2360	+	Rae1		9.4e-32	
Tb10.6k15.2350	+	Nup155		5.4e-24	
Tb11.02.0460	+	Nup107		0.02	
Tb10.6k15.3670	+	Nup93		0.0012	
Tb1927.4.2880	+	Nup205		0.96	
Tb11.03.0140	+	Nup96/98		0.0012	
Tb11.01.7200	+	Nup62		8.2e-05	
Tb927.4.5200	+	Nup54		1.2e-08	
Tb927.3.3180	−	−	+	0.016	FG repeats
Tb927.3.3540	−	−	+	2.1	FG repeats
Tb11.02.0270	−	−	+	0.16	FG repeats
Tb11.01.2880	−	−	+	1.1	FG repeats
Tb927.4.4310	−	−	+	3.4	FG repeats
Tb927.8.8050	−	−	+	4	FG repeats
Tb11.01.2885	−	−	+	0.0009	FG repeats
Tb11.03.0810	−	−	+	5.8	
Tb10.6k15.1530	−	−	−	ND	
Tb09.211.4780	−	−	−	ND	
Tb09.160.0340	−	−	−	ND	
Tb11.01.7630	−	−	−	ND	
Tb927.7.2300	−	−	−	ND	

a Sequence present in HMMer hit list but excluded due to weak similarities (e.g. restricted FG repeats) to known Nups.

b ND: Sequence not detected in HMMer-based screen.

### Components from all NPC subcomplexes are present in LECA

The results of our full screen for Nups are summarised in Figure 1, with species-level detail given in Table 2 (accession numbers for candidates are given in supplementary Table S1). We report that homologs for 19 of 31 Nups are found in all five supergroups (Fig. 1), significantly extending the findings of previous studies, which were based on the analysis of fewer genomes [10], [11].

**Table 2:** Distribution of candidate nucleoporins identified using HMMer*.

		Opisthokonts																												Amoebozoa			Plantae							Chromalveolates								Excavates				
		Dipterans			Vertebrates							Tunicates	Nematodes	Choanoflagellates	Ascomycetes												Basidiomycetes		Microsporidia			Rhodophyte	Chlorophytes			Streptophytes				Ciliate	Heterokonts			Apicomplexans				Diplomonads	Heterolobosea	Parabasalids	Kinetoplastids	
		***Ag***	***Am***	***Dm***	***Hs***	***Mm***	***Rn***	***Gg***	***Xl***	***Dr***	***Tn***	*Ci*	***Ce***	*Mb*	***Sc***	***Kl***	*Ps*	***Cg***	***Ca***	***Dh***	***Yl***	*Nc*	***Gz***	*An*	***Mg***	*Sp*	*Um*	*Cn*	*Ec*	*Eh*	*Dd*	***Cm***	*Vc*	*Ot*	*Cr*	*Pp*	*Pt*	***Os***	***At***	*Tt*	*Ptr*	*Pi*	*Tps*	*Tp*	*Ch*	*Tg*	*Pb*	*Gl*	*Ng*	*Tv*	*Tb*	*Lm*
***LOCALIZATION***	***NUP***																																																			
	**Gle1**	x	x	x	x	x	x	x		x	x	x		x	x	x	x	x	x	x	x	x	x	x	x	x	x	x			x	x		x	x	x	x	x	x		x	x	x		x				x			
	**Aladin**	x		x	x	x	x	x	x	x	x	x		x																	x	x	x	x		x	x	x	x			x							x		x	x
**Cytoplasmic fibrils**	**Nup88**	x	x	x	x	x	x	x	x	x	x	x			x	x	x	x	x	x	x	x	x	x	x	x	x	x			x	x	x	x	x	x	x	x	x			x										
	**Nup214**	x	x	x	x	x	x	x	x	x	x	x	x	x	x	x	x	x	x	x	x	x	x	x	x	x	x	x			x			x			x	x	x			x				x			x			x
	**Nup358**	x	x	x	x	x	x	x	x	x	x	x	x	x																																						
**Scaffold**	**Nup160**	x	x	x	x	x	x	x	x	x	x	x	x	x	x	x	x	x	x	x	x	x	x	x	x	x	x	x			x		x	x		x	x	x	x			x							x			
	**Nup133**	x	x	x	x	x	x	x		x	x	x	x		x	x	x	x	x	x	x	x	x	x	x	x	x	x			x	x	x	x	x	x	x	x	x		x	x	x						x			
	**Nup107**	x	x	x	x	x	x	x	x	x	x	x	x	x	x	x	x	x	x	x	x	x	x	x	x	x	X	x			x	x	x	x		x	x	x	x		x	x	x					x	x	x	x	x
	**Nup75**	x	x	x	x	x	x	x	x	x	x	x	x	x	x	x	x	x	x	x	x	x	x	x	x	x	x	x			x		x	x	x	x	x	x	x			x							x			
	**Nup43**	x	x	x	x	x	x	x	x	x	x	x	x	x																	x					x	x	x	x			x										
	**Nup37**	x	x	x	x	x	x	x	x	x	x	x									x	x	x	x	x	x																										
	**Seh1**	x	x	x	x	x	x	x	x	x	x	x	x	x	x	x	x	x	x	x	x			x		x		x			x	x	x	x	x	x	x	x	x	x	x	x							x		x	x
	**Sec13**	x	x	x	x	x	x	x	x	x	x	x	x	x	x	x	x	x	x	x	x	x	x	x	x	x	x	x	x	x	x	x	x	x	x	x	x	x	x	x	x	x	x		x	x	x	x	x	x	x	x
	**Nup205**	x	x	x	x	x	x	x	x	x	x	x	x	x	x	x	x	x	x	x	x	x	x	x	x	x	x	x					x	x	x	x	x	x	x			x							x	x	x	x
	**Nup188**	x	x	x	x	x	x	x	x	x	x	x	x		x	x	x	x	x	x	x	x	x	x	x	x	x	x					x	x	x	x	x	x	x													
	**Nup155**	x	x	x	x	x	x	x	x	x	x	x	x	x	x	x	x	x	x	x	x	x	x	x	x	x	x	x	x		x	x	x	x	x	x	x	x	x	x	x	x	x		x			x	x	x	x	x
	**Nup93**	x	x	x	x	x	x	x	x	x	x	x	x	x	x	x	x	x	x	x	x	x	x	x	x	x	x	x	x		x	x	x		x	x	x	x	x	x	x	x	x						x	x	x	x
	**Nup35**	x	x	x	x	x	x	x	x	x	x	x	x	x	x	x	x	x	x	x	x					x		x	x		x	x	x	x	x	x	x	x	x		x	x	x					x		x		
**Transmembrane**	**Ndc1**	x		x	x	x	x	x	x	x	x	x	x		x	x	x	x	x	x	x	x	x	x	x	x	x	x						x	x	x	x	x	x			x								x		
	**Pom121**				x	x	x	x	x	x	x																																									
	**Gp210**	x	x	x	x	x	x	x	x	x	x	x	x	x																x	x					x	x	x	x	x				x	x	x			x	x		
	**Pom152**														x	x	x	x	x	x	x	x	x	x	x	x	x	x																								
	**Pom34**														x	x	x	x	x	x	x	x	x	x	x	x																										
**Nuclear ring**	**Nup96-98**	x		x	x	x	x	x	x	x	x	x	x	x	x	x	x	x	x	x	x	x	x	x	x	x	x	x	x	x	x	x	x	x	x	x	x	x	x	x	x	x	x	x	x	x	x	x	x	x	x	x
	**Rae1**	x	x	x	x	x	x	x	x	x	x	x	x	x	x	x	x	x	x	x	x	x	x	x	x	x	x	x	x	x	x	x	x	x	x	x	x	x	x	x	x	x	x	x	x	x		x	x		x	x
**Central**	**Nup62**	x	x	x	x	x	x	x	x	x	x	x	x	x	x	x	x	x	x	x	x	x	x	x	x	x	x	x	x	x	x	x	x	x		x	x	x	x		x	x	x		x	x			x	x	x	x
**channel**	**Nup58**	x	x	x	x	x	x	x	x	x	x	x		x	x	x	x	x	x	x	x	x	x	x	x	x	x	x			x		x		x	x	x	x	x			x							x			x
	**Nup54**	x	x	x	x	x	x	x	x	x	x	x	x	x	x	x	x	x	x	x	x	x	x	x	x	x	x	x			x	x	x	x	x	x	x	x	x	x	x	x	x			x		x	x	x	x	x
**Nuclear**	**Nup50**	x	x	x	x	x	x	x	x	x	x	x	x	x	x	x	x	x	x	x	x	x	x	x	x	x	x	x			x			x			x	x	x		x	x	x						x			
**basket**	**Nup153**		x	x	x	x	x	x	x	x	x	x	x		x	x	x	x	x	x	x					x	x	x						x			x	x	x		x	x	x						x			
	**TPR**	x	x	x	x	x	x	x	x	x	x	x	x	x	x	x		x	x	x	x	x	x	x	x	x	x	x			x		x	x		x	x	x	x		x	x	x						x			
**Nups in supergroup**		**31**																												**22**		**26**								**25**								**23**				

*Species names (note bold font indicates species from which training dataset is derived): Ag:*Anopheles gambiae,* Am:*Apis mellifera,* Dm:*Drosophila melanogaster,* Hs:*Homo sapiens,* Mm:*Mus musculus,* Rn:*Rattus norvegicus,* Gg:*Gallus gallus,* Xl:*Xenopus laevis,* Dr:*Danio rerio,* Tn:*Tetraodon nigroviridis,* Ci:*Ciona intestinalis,* Ce:*Caenorhabditis elegans,* Sc:*Saccharomyces cerevisiae,* Kl:*Klyveromyces lactis,* Ps:*Pichia stipitis,* Cg:*Candidata glabrata,* Ca:*Candida albicans,* Dh:*Debaromyces hansenii,* Yl:*Yarrowia lipolytica,* Nc:*Neurospora crassa,* Gz:*Giberella zeae,* An:*Aspergillus nidulans,* Mg:*Magnaporthe grisea,* Sp:*Schizosaccharomyces pombe,* Um:*Ustilago maydis,* Cn:*Cryptococcus neoformans,* Ec:*Encephalitozoon cuniculi,* Mb:*Monosiga brevicollis,* Eh:*Entamoeba histolytica,* Dd:*Dictyostelium discoideum,* Cm:*Cyanidioschizon merolae,* Vc:*Volvox carteri,* Ol:*Ostreococcus lucimarinus,* Cr:*Chlamydomonas reinhardtii,* Pp:*Physcomitrella patens,* Pt:*Populus trichocarpa,* Os:*Oryza sativa,* At:*Arabidopsis thaliana,* Tt:*Tetrahymena thermophila,* Ptr:*Phaeodactylum tricornutum,* Pi:*Phytophthora infestans,* Tps:*Thalassiosira pseudonana,* Tp:*Theileria parva,* Ch:*Cryptosporidium hominis,* Tg:*Toxoplasma gondii,* Pb:*Plasmodium berghei,* Gl:*Giardia lamblia,* Ng:*Naegleria gruberi,* Tv:*Trichomonas vaginalis,* Tb:*Trypanosoma brucei, Lm:Leishmania major.*

The broadest conservation is found in plants where we detect 26 candidates, suggesting that the core composition of nuclear pore complexes in green plants is highly similar to that seen among opisthokonts. The only genome within the Plantae for which no Nups were recovered is the nucleomorph genome of *Hemiselmis andersenii*, which derives from a red algal endosymbiont [35], [36]. This result mirrors previous results indicating that the nucleomorph genomes of *Guillardia theta* and *Bigelowiella natans* are devoid of nucleoporin genes, suggesting that all nucleoporin genes are coded in the main nucleus instead [9]. That all available nucleomorph genomes lack obvious nucleoporin homologs suggests little hindrance to relocation or replacement of nucleoporin genes in these lineages.

Candidate nucleoporins were also readily identified in Amoebozoa (22), Chromalveolates (25) and Excavates (23) (Figure 1, Table 2), with nucleoporins from all key subcomplexes and substructures (cytoplasmic fibrils, scaffold, anchoring system, nuclear ring, central channel and nuclear basket) being detected in members of all supergroups.

In previous studies, the conclusion that the LECA possessed a NPC was complicated by the patchy distribution of some nucleoporins, with only 9 nucleoporins found in any of the supergroups other than Plantae and Opisthokonts [11] [10]. Consequently, the ability to assign a complex NPC to LECA differed depending upon the topology of the eukaryote tree; where Excavates were basal (see [37]), only 7 nucleoporins could be placed in LECA [10]. If the root was placed between unikonts and bikonts [38], [39], 23 Nups could be traced back to LECA, largely on account of candidates identified in plants [10], [11]. As shown in Table 3, our broad screen significantly expands the extent to which Nup homologues can be identified across the eukaryote tree. Our results increase the number of Nup candidates across all eukaryote supergroups where genome data are available (except Opisthokonts, where a full complement had already been characterised in advance of all three studies). Of particular note, we significantly expand the number of candidates in three eukaryote supergroups where genome sequence data is still limited (Amoebozoa, Chromalveolates and Excavates). For these supergroups our screen expands the total number of candidate Nups from fewer than ten in each supergroup to 22 in Amoebozoa 25 in Chromalveolates and 23 in Excavates (Table 3).

**Table 3:** Comparison of Nup identification in the present study with previously published screens*.

		Opisthokonts			Amoebozoa			Plantae			Chromalveolates			Excavates		
		N	M	B	N	M	B	N	M	B	N	M	B	N	M	B
	**Gle1**	x	x	x	x			x	x		x	x	x	x		
	**Aladin**	x	x	x	x		x	x	x	x	x			x	x	
**Cytoplasmic fibrils**	**Nup88**	x	x	x	x		x	x	x	x	x		x			
	**Nup214**	x	x	x	x			x			x			x		
	**Nup358**	x	x	x												
**Scaffold**	**Nup160**	x	x	x	x			x	x	x	x			x		
	**Nup133**	x	x	x	x			x	x	x	x			x		
	**Nup107**	x	x	x	x			x	x	x	x			x		
	**Nup75**	x	x	x	x		x	x	x	x	x		x	x		
	**Nup43**	x	x	x	x			x	x	x	x					
	**Nup37**	x	x	x						x						x
	**Seh1**	x	x	x	x	x	x	x	x	x	x		x	x		x
	**Sec13**	x	x	x	x	x	x	x	x	x	x	x	x	x	x	x
	**Nup205**	x	x	x			x	x	x	x	x			x		
	**Nup188**	x	x	x				x	x	x						
	**Nup155**	x	x	x	x	x		x	x	x	x	x	x	x	x	x
	**Nup93**	x	x	x	x			x	x	x	x			x		
	**Nup35**	x	x	x	x			x	x	x	x			x	x	x
**Transmembrane**	**Ndc1**	x	x	x				x			x			x		
	**Pom121**	x	x	x												
	**Gp210**	x	x	x	x			x	x		x	x		x		
	**Pom152**	x	x	x												
	**Pom34**	x	x	x												
**Nuclear ring**	**Nup96-98**	x	x	x	x	x	x	x	x	x	x	x		x	x	
	**Rae1**	x	x	x	x	x	x	x	x	x	x	x	x	x	x	x
**Central channel**	**Nup62**	x	x	x	x	x	x	x	x	x	x			x	x	
	**Nup58**	x	x	x	x			x		x	x			x		
	**Nup54**	x	x	x	x			x	x	x	x			x		
**Nuclear basket**	**Nup50**	x	x	x	x			x		x	x			x		
	**Nup153**	x	x	x				x			x			x	x	
	**TPR**	x	x	x	x			x	x	x	x			x		
**Nups in supergroup**		**31**	**31**	**31**	**22**	**6**	**9**	**26**	**21**	**22**	**25**	**6**	**7**	**23**	**8**	**6**

*N: this study; M: Mans et al. [10]; B: Bapteste et al. [11]. N.B. This table aims simply to show that our updated screen now enables a more confident assignment of a complete NPC to the LECA than was possible based on two earlier studies. We have not performed a systematic comparison of the different methods applied across the three studies. This table therefore does not directly compare an HMM-based approach with PSI-BLAST or blast with ancestral sequence reconstruction (such comparisons exist, e.g. [100]). Moreover, the present study screened additional genome sequences unavailable at the time the other studies were performed. As is evident from Table 2, screening of a number of recently published eukaryote genomes has contributed greatly to a more complete reconstruction of the NPC in the LECA.

**Figure 2:**
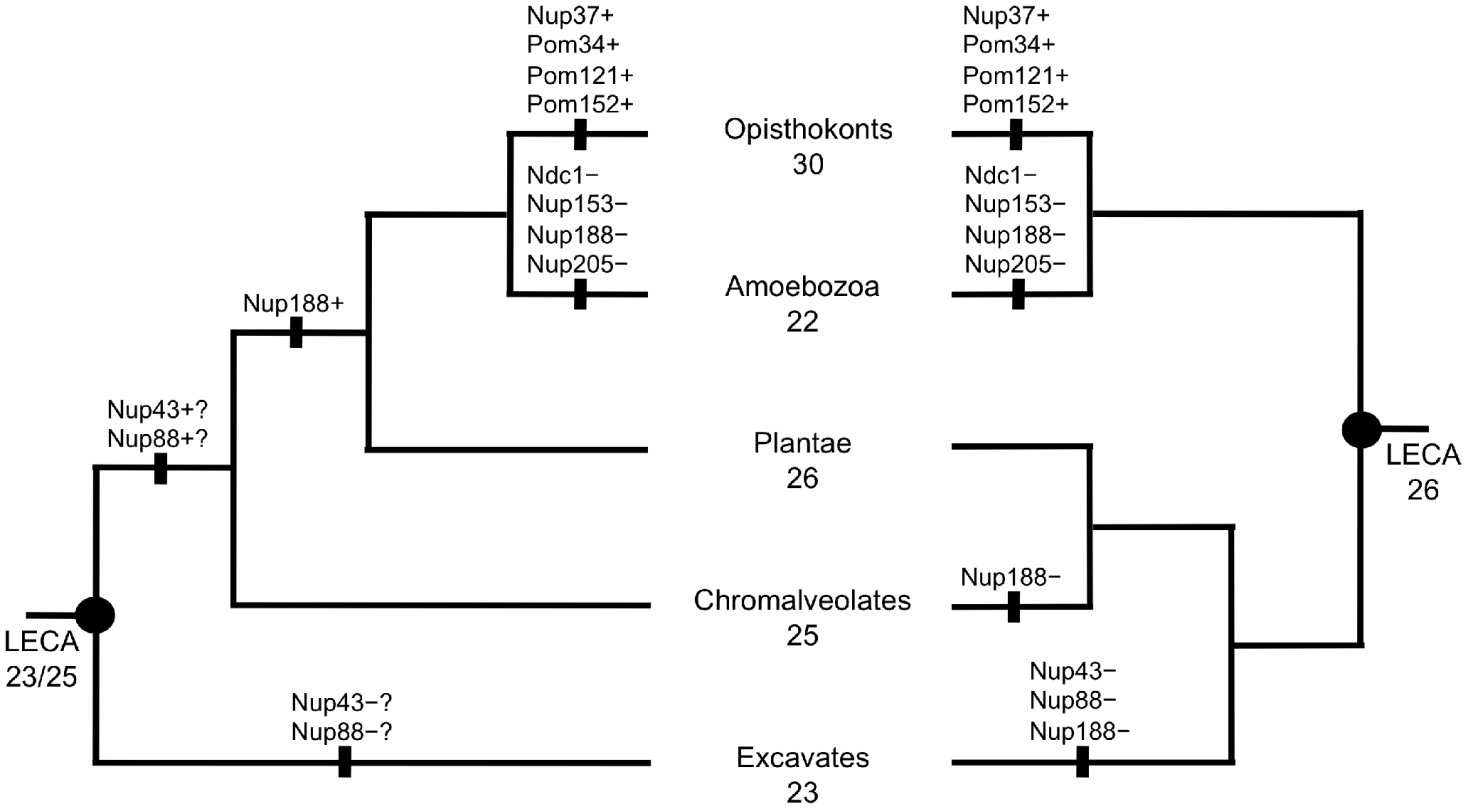
NPC components are traceable to LECA. NPC pore composition in LECA based on two alternative rootings of the eukaryote tree. In the left hand tree, Excavates are the outgroup. The right hand tree is rooted on the basis of the unikont/bikont bifurcation. Gains (+) and losses (–) in different lineages are indicated under each scenario. Where gains and losses are equally probable, these are marked with (?).

The identification of so many new Nup candidates across Amoebozoa, Chromalveolates and Excavates is significant because it enables us to trace a complex NPC back to LECA regardless of ongoing uncertainty about the position of the root of the eukaryote tree (Figure 2), thereby providing robust evidence for the early evolutionary origin of the NPC in the eukaryote lineage independently of tree topology. By contrast, previous studies could only unambiguously place a complex NPC in the common ancestor of Opisthokonts and the Plantae. Under the unikont/bikont rooting (Figure 2, right tree), we can trace 26 nucleoporins back to LECA (Fig 2), with four gains in the Opisthokonts. Of these, three are clearly lineage-specific gains: Pom121 is restricted to vertebrates, while Pom34 and Pom152 are found only in fungi. Nup37 is found in metazoa and some ascomycetes, suggesting either that we have failed to find all orthologs, or that this Nup has been subject to a series of losses in the Opisthokonts — the recent identification of a Nup37 homolog in *Aspergillus nidulans* [40] confirms these ascomycete candidates are not spurious predictions.

It is likewise interesting that Amoebozoa appear from Figure 2 to have lost a number of Nups. However losses (as indicated on both trees in Figure 2) should be treated with caution in that it is difficult to distinguish between genuine loss and missing data. In this context, it will be interesting to analyse genome data from the anaerobic amoebozoan, *Breviata anathema*, which is proposed to represent a deep-branching member of this supergroup [41], [42].

A cursory examination of Table 2 indicates that we have had only limited success in finding Nup candidates among some parasitic lineages, and observations supporting morphologically complex nuclear pores among excavates [32], [43], underscore the necessarily conservative nature of comparative genomic analyses. That aside, the data nevertheless provide a clear indication that LECA possessed between 23 and 26 Nups. Given ongoing uncertainty concerning the structure of the eukaryote tree [42], [44], we note that, assuming the genomes screened in the present study are correctly placed in the proposed five supergroups, a star tree would still suggest between 19 and 22 Nups in LECA (where 19 are found in at least one representative genome from each supergroup and 22 is the minimum number of Nups in any one supergroup — Figure 1). That all major subcomplexes are represented even in the most conservative estimate (19 Nups) suggests LECA possessed a NPC comparable in complexity to NPCs in modern eukaryotes.

### Evidence for a rudimentary NPC anchoring system in LECA

While the NPC does not traverse the lipid bilayer of either the inner our outer nuclear membrane, several nucleoporins are involved in anchoring the NPC to the nuclear envelope (reviewed in [2], [6]). Among characterised Nups involved in anchoring, Pom34 and Pom152 are thought to be restricted to fungi, whereas Pom121 and Gp210 are vertebrate-specific (reviewed in [40]). The apparent lack of overlap led to the suggestion that the anchoring system may either be restricted to opisthokonts, or that it evolved by convergence [11]. Ndc1, a known transmembrane Nup from yeast, has recently been demonstrated to be a constituent of a range of fungal and vertebrate NPCs [45], [46], [47], indicating that parts of the anchoring system evolved before the split of vertebrates and fungi.

Our results significantly extend this view (Figure 1 & Table 2). We identify homologs for Gp210 across all five supergroups, with multiple candidates across Amoebozoa, Plants, Chromalveolates and Excavates. It therefore seems probable that the absence of Gp210 from Fungi, where constituent Nups have been extensively characterised [48], is the result of secondary loss. Identification of Ndc1 homologs is somewhat more restricted; it is readily detected in green algae and plants (Table 2), but only a single candidate is detected among the Chromalveolates (*Phytophthora infestans*), likewise among Excavates (*Trichomonas vaginalis*), and we found no candidates among the Amoebozoa. As shown in Figure 2, the distribution of Ndc1 nevertheless suggests this Nup can be placed in LECA, under either rooting. Splitstree analyses showed the Ndc1 dataset was noisy; a simple distance-based tree (BioNJ, JTT, γ, 100 bootstrap replicates) does not indicate recent horizontal gene transfer from either Plantae or Opisthokonts to either of these lineages (Supplementary Figure S1).

Bolstering the suggestion that LECA possessed an anchoring system is the broad distribution of Nup35 (known as Nup53 in yeast and some vertebrates [6]), which is also conserved across all five supergroups. Nup35 is integral to NPC assembly [49], [50], it interacts directly with Ndc1 [47], [49] and may also contribute to anchoring of the NPC to the nuclear envelope via an amphipathic α-helix [51]. We therefore suggest that Gp210 and Ndc1, possibly with the inclusion of Nup35, constitute the ancestral anchoring system in LECA.

### Does the distribution of integral membrane Nups shed any light on the evolution of variant mitoses?

In stark contrast to the results for Gp210, Ndc1 and Nup35, the other integral membrane Nups (Pom34, Pom121 & Pom152) display a more limited distribution (Table 2). It has been noted that the non-overlapping distribution of these three transmembrane Nups correlates with open mitoses in vertebrates (Pom121) and closed mitoses in yeasts (Pom34 and Pom152) [12]. In closed mitosis, the nuclear envelope remains intact during cell division, whereas in open mitosis, the nuclear envelope disintegrates, and envelope and NPC must be reassembled following division [52], though there appear to be many variations therein [13], [53]. Stunningly, experimental studies have demonstrated partial disassembly of the NPC during so-called ‘closed’ mitosis in *Aspergillus nidulans* [54]. However, Pom152 remains associated with the nuclear envelope. In a *Saccharomyces cerevisiae pom34ΔN nup188Δ* double mutant, Miao et al. [12] observed disassembly of some of the same FG repeat-containing Nups as were disassociated during closed mitosis in *A. nidulans*, raising the possibility that both may be central to (partial) pore maintenance during closed mitoses.

While a degree of caution is warranted concerning the open/closed mitosis dichotomy [53], particularly among the Fungi (but also in early development in *Drosophila* and *Caenorhabditis* species, where early embryonic nuclei divide in a syncytium), our data do shed some light on the correlations noted by Miao et al. [12].

In the case of animals, it seems that Pom121 is restricted to vertebrates (Table 2): we find no homologs of Pom121 in diptera, tunicate or nematode genomes analysed, nor do we find a candidate in *Monosiga brevicollis*, a Choanoflagellate (sister group to metazoa — [55]; all of these groups undergo open mitoses [13], [56]). On these data, it seems difficult to assign a general role for Pom121 in open mitosis, though a specific role in this process in vertebrates is of course plausible [57].

A more informative picture emerges across the fungal genomes however. We note that Ascomycetes as a group are characterised by closed mitoses [13], whereas among Basidiomycetes no cases of closed mitosis have been reported, and open mitoses are well-characterised in a number of species (reviewed in [58]).

Our initial analyses (Table 2) indicated that Pom152 was present across all fungi, but no Pom34 homologs were present in the two Basidiomycetes included in our screen, *Ustilago maydis* & *Cryptococcus neoformans*, both of which exhibit open mitosis [58], [59]. To further examine this pattern, we screened four additional Basidiomycete genomes (*Phanerochaete chrysosporium, Laccaria bicolour, Coprinossis cinea* & *Malassesezia globosa*) as well as that of the zygomycete *Rhizopus oryza*, which is thought to likewise undergo open mitosis [58]. As is clear from Table 4, all fungal genomes screened carry both Ndc1 and Pom152 homologs, but Pom34 is restricted to Ascomycetes. Given the broad phylogenetic distribution of ascomycete species included in our analysis [60], it seems reasonable to conclude that Pom34 was present in the ancestor of this group, but not in that of Basidiomycetes as suggested by the complete absence of Pom34 homologs among those fungi.

**Table 4:** Distribution of anchoring nucleoporins across Fungi*.

		‘Closed’ mitosis												‘Open’ mitosis						
		Ascomycetes												Basidomycetes						Zygomycetes
		*An*	*Yl*	*Ps*	*Nc*	*Sp*	*Sc*	*Kl*	*Ca*	*Cg*	*Dh*	*Gz*	*Mg*	*Cn*	*Pc*	*Um*	*Lb*	*Cc*	*Ml*	*Ro*
**Nucleoporin**	**Ndc1**	x	x	x	x	x	x	x	x	x	x	x	x	x	x	x	x	x	x	x
	**Pom152**	x	x	x	x	x	x	x	x	x	x	x	x	x	x	x	x	x	x	x
	**Pom34**	x	x	x	x	x	x	x	x	x	x	x	x							

*Species abbreviations: *An: Aspergillus nidulans, Yl: Yarrowia lipolytica, Ps: Pichia stipitis, Nc: Neurospora crassa, Sp: Schizosaccharomyces pombe, Sc: Saccharomyces cerevisiae, Kl: Kluyveromyces lactis, Ca: Candida albicans, Cg: Candida glabrata, Dh: Debaryomyces hansenii, Mg: Magnaporthe grisea, Cn: Crytpococcus neoformans, Pc: Phanerochaete chrysosporium, Um: Ustilago maydis, Lb: Laccaria bicolor, Cc: Coprinossis cinea, Ml: Malassezia globosa, Ro: Rhizopus oryzae.*

This result suggests that Pom34, but not Pom152, is central to this distinction, at least within dikaryote fungi. We failed to find evidence of either Pom34 or Pom152 in the microsporidian *Encephalitozoon cuniculi*, which undergoes closed mitosis [61], indicating that if Pom34 is integral to the evolution of closed mitosis in Fungi, this may only be limited to Ascomycetes. Having said that, only seven Nups were detected in *E. cuniculi*, and the combination of reductive adaptation to a parasitic lifestyle and rapid sequence-level evolution for some genes [62] may complicate homolog detection in this lineage. In this respect, it does seem that at least part of the anchoring system may well have evolved multiple times [11]. In that there appears to be a spectrum between open and closed forms of mitosis [53], and given that open and closed mitoses likely have a complex evolutionary history [13] [63], experimental screens may well yield a broader diversity of pore membrane (POM) proteins than hitherto recognised.

### Complete coatomer complex components are traceable to LECA

The observation that Nups and coatomer proteins share a common architecture [18], [64] has led to the proposal that these also share a common evolutionary origin. This protocoatomer hypothesis [18] is supported by the observation that vesicle coat proteins are well conserved across eukaryotes [65], [66], [67], [68] and have expanded via duplication and divergence [67], [69], [70]. Vesicle coat complexes are involved in movement of cargo between the various organelles that constitute the endomembrane system, and are one part of this evolutionarily conserved system that also includes the evolutionarily ancient but distinct ESCRT system [67], [71].

While previous analyses leave little doubt that the COPI, COPII, clathrin/adaptin complexes, are a feature of LECA, less focus has been placed on patterns of conservation at the level of individual components. We therefore screened for individual protein subunits from each complex across a representative dataset spanning five supergroups. In contrast to the overall pattern of conservation of the NPC, the COPI, II and clathrin/AP complexes were extremely well conserved and orthology predictions were assessable using phylogenies (see Supplementary file SI4). At the level of supergroups there are only four discernible differences (Table 5; accession numbers are in supplementary Table S2). Apm2, a clathrin adaptor protein medium (µ)-chain protein homolog, appears restricted to Saccharomycetes, and can be readily attributed to gene duplication (supplementary Figure S2). However, it remains unclear whether Apm2 is a bona fide component of Clathrin complexes. Data to date indicate no discernible phenotype in yeast knockouts [72], it has not been ascribed to any AP complexes in yeast [73], [74], and interaction with Apl2p (a constituent of the AP-1 clathrin adaptor complex) is only clearly observed when Apm2p is overexpressed [75].

**Table 5:** Distribution of candidate coatomer complex components across eukaryotes*.

		Opisthokonts																								Amoebozoa		Plantae							Chromalveolates									Excavates			
		Diptera		Vertebrates							Tunicates	Nematodes	Choanoflagellates	Ascomycetes									Basidiomycetes		Microsporidia			Rhodophytes	Chlorophytes			Streptophytes			Ciliates	Heterokonts			Apicocomplexa					Diplomonads	Heterolobosae	Parabasalids	Kinetoplastids
		***Am***	***Dm***	***Hs***	***Mm***	***Rn***	***Gg***	***Bt***	***Xl***	***Dr***	***Ci***	***Ce***	***Mb***	***Sc***	***Ps***	***Cg***	***Ca***	***Dh***	***Yl***	***Nc***	***An***	***Sp***	***Um***	***Cn***	***Ec***	***Eh***	***Dd***	***Cm***	***Vc***	***Ot***	***Cr***	***Pp***	***Os***	***At***	***Tt***	***Ptr***	***Pi***	***Tps***	***Tp***	***Ch***	***Tg***	***Pf***	***Pb***	***Gl***	***Ng***	***Tv***	***Li***
**Complex**	**Subunit**																																														
**COPI**	**α**	x	x	x	x	x	x	x	x	x	x	x	x	x	x	x	x	x	x	x	x	x	x	x	x	x	x	x	x	x	x	x	x	x	x	x	x	x	x	x	x	x	x	x	x	x	x
	**β**	x	x	x	x	x	x	x	x	x	x	x	x	x	x	x	x	x	x	x	x	x	x	x	x	x	x	x	x	x	x	x	x	x	x	x	x	x	x	x	x	x	x	x	x	x	x
	**β**'		x	x	x	x	x	x	x	x	x	x	x	x	x	x	x	x	x	x	x	x	x	x	x	x	x	x	x	x	x	x	x	x	x	x	x	x	x	x	x	x	x	x	x	x	x
	**γ**	x	x	x	x	x	x	x	x	x	x	x	x	x	x	x	x	x	x	x	x	x	x	x	x	x	x	x	x	x	x	x	x	x	x	x	x	x	x	x	x	x	x		x	x	x
	**δ**	x	x	x	x	x	x	x	x	x	x	x	x	x	x	x	x	x	x		x	x	x	x	x	x	x	x	x	x	x	x	x	x	x	x	x	x	x	x	x	x	x	x	x	x	x
	**ε**	x	x	x	x	x	x	x	x	x	x	x	x	x	x	x	x	x	x	x	x		x	x		x	x	x	x	x	x	x	x	x	x	x	x	x			x				x	x	x
	**ζ**	x	x	x	x	x		x	x	x	x	x	x	x	x	x	x	x	x	x	x	x	x	x	x	x	x	x	x	x	x	x	x	x	x	x	x	x	x	x	x	x	x	x	x	x	x
**COPII**	**sar1**	x	x	x	x	x	x	x	x	x	x		x	x	x	x	x	x	x	x	x	x	x	x	x	x	x	x	x	x	x	x	x	x	x	x	x	x	x	x	x	x	x	x	x	x	x
	**sec16**	x	x	x	x	x	x	x	x	x	x		x	x	x	x	x	x	x	x	x	x	x	x						x	x	x	x	x		x	x								x		
	**sec23**	x	x	x	x	x	x	x	x	x	x	x	x	x	x	x	x	x	x	x	x	x	x	x	x	x	x	x	x	x	x	x	x	x	x	x	x	x	x	x	x	x	x	x	x	x	x
	**sec24**	x	x	x	x	x	x	x	x	x	x	x	x	x	x	x	x	x	x	x	x	x	x	x	x	x	x	x	x	x	x	x	x	x	x	x	x	x	x	x	x	x	x	x	x	x	x
	**sec31**	x	x	x	x	x	x	x	x	x	x	x	x	x	x	x	x	x	x	x	x	x	x	x	x	x	x	x	x	x	x	x	x	x	x	x	x	x	x	x	x	x	x	x	x	x	x
	**sfb3**	x	x	x	x	x	x	x	x	x	x		x	x		x	x	x	x	x	x	x	x		x	x	x				x	x	x	x													
**Clathrins & clathrin- associated adapter complex components**	**chc1**	x	x	x	x	x	x	x	x	x	x	x	x	x	x	x	x	x	x	x	x	x	x	x		x	x	x	x	x	x	x	x	x	x	x	x	x	x	x	x	x	x	x	x	x	x
	**clc1**	x	x	x	x	x	x	x	x	x	x	x		x	x	x	x	x	x	x	x	x	x	x			x		x	x	x	x	x	x	x	x	x	x			x				x	x	x
	**apl1**			x	x	x	x	x	x	x				x	x	x	x	x	x	x	x	x		x																							
	**apl2**		x	x	x	x		x	x	x	x	x		x	x	x	x	x	x	x			x		x	x	x	x		x	x	x	x	x	x	x	x	x	x	x	x	x	x	x	x	x	x
	**apl3**	x	x	x	x	x	x	x	x	x	x	x	x	x	x	x	x	x	x	x	x	x	x	x		x	x		x	x	x	x	x	x	x	x	x	x	x			x	x	x	x	x	x
	**apl4**		x	x	x	x	x	x	x	x	x	x	x	x	x	x	x	x	x		x	x	x	x		x	x	x	x	x	x	x	x	x		x	x	x	x	x	x	x	x	x	x	x	x
	**apl5**	x	x	x	x	x		x	x	x	x	x	x	x	x	x	x	x	x	x	x	x	x	x		x	x	x		x		x	x	x		x	x	x			x				x	x	x
	**apl6**	x	x	x	x	x		x	x	x	x	x	x	x	x	x	x	x	x	x	x	x	x	x			x			x		x	x	x		x	x	x			x	x	x		x	x	x
	**apm1**	x	x	x	x	x	x	x	x	x	x	x	x	x	x	x	x	x	x	x	x	x		x	x	x	x	x	x	x	x	x	x	x	x	x	x	x	x	x	x	x	x		x	x	x
	**apm3**	x	x	x	x	x	x	x	x	x	x	x	x	x	x	x	x	x	x	x	x	x	x	x		x	x			x		x	x	x	x	x	x	x		x	x			x	x	x	x
	**apm4**	x	x	x	x	x	x	x	x	x	x	x	x	x	x	x	x	x	x	x	x	x	x	x		x	x		x	x	x	x	x	x	x	x	x	x			x	x	x	x	x	x	x
	**aps1**	x	x	x	x	x	x	x	x	x		x	x	x	x	x	x	x		x	x	x	x	x		x	x	x	x	x	x	x	x	x	x	x		x	x	x	x	x	x		x	x	x
	**aps2**	x	x	x	x	x		x	x	x	x	x	x	x	x	x	x	x	x	x	x	x		x		x	x		x	x	x	x	x	x	x	x	x	x	x		x	x	x	x	x	x	x
	**aps3**	x	x	x	x	x	x	x	x	x	x	x	x	x	x	x	x	x	x	x	x	x	x	x	x	x	x	x		x		x	x	x		x	x	x			x	x	x		x	x	x

*Abbreviations as per Table 2, with the addition of Bt: *Bos taurus*, Pf: *Plasmodium falciparum* and Li*: Leishmania infantum*. Training data for HMMs derived from PSI blast outputs.

Vertebrate Apl1 has likewise clearly evolved via duplication from the more broadly distributed Apl2 (supplementary Figure S3). Fungi also contain both Apl1 and Apl2, but these form distinct phylogenetic clans (Figure S3), suggesting fungal Apl1 and Apl2 are paralogues that did not evolve via duplication in an early fungal lineage. Non-Ophisthokont Apl2 sequences appear to form two separate clans in the unrooted tree inconsistent with eukaryote supergroups, suggesting that Apl2 and fungal Apl1 have evolved via a complex pattern of ancient duplications and losses. The trees are not sufficiently robust to establish all events with confidence, but a robust minimal conclusion is that vertebrate and fungal Apl1 have separate evolutionary origins.

We find only two other instances where an entire supergroup lacks a component; both impact COPII: the two amoebozoa represented here (*Entamoeba histolytica* and *Dictyostelium discoideum*) lack Sec16, a COPII constitutent, but in contrast to previous analyses [65] we do find candidates for all other COPII components in this group. The other supergroup-level absence is Sfb3, for which no homologs were recovered from either Excavates or Chromalveolates. In *S. cerevisiae*, Sfb3 is involved in vesicle budding and transport of cargo from the ER but not vesicle fusion with the Golgi body. Its function can be compensated for at lower temperatures by Sec24, with which it is homologous [76]. We identified Sec24 homologs in all Excavate and Chromalveolate genomes we screened, so in a scenario where Excavates and Chromalveolates represent the deepest branches of the eukaryote tree (as per Figure 2, left hand tree), the only innovation since LECA would be a single gain of a duplicate gene in the lineage leading to Plantae, Amoebozoa and Opisthokonts. Under the Unikont/Bikont rooting (cf Figure 2, right hand tree), this ‘innovation’ vanishes and is instead two losses. That such extreme conservation of components exists at the supergroup level is stunning.

### WD-repeats are present in Bacteria and Archaea

Previous analyses report the presence of weak homologs to NPC components in bacteria and archaea, though no published data point to nuclear pore complex constituents in the genomes of either domain [10], [11]. Supplementary Table S3 summarizes the results of our HMMer-based screen as applied to 49 bacterial and archaeal genomes. We found numerous hits in both archaea and bacteria to WD-repeat containing proteins. WD-repeat proteins possess a characteristic β-propellor fold [77] and are important for protein binding as they can form reversible complexes with several proteins, allowing coordination of sequential and/or simultaneous interactions that involve several sets of proteins at the same time. They comprise a large family involved in a variety of essential biological functions such as signal transduction, transcription regulation and apoptosis [78]. While to our knowledge no WD-repeat proteins have been characterized in Archaea, a small number have been characterized in bacteria, including AglU, which is required for gliding motility and development of spores in *Myxococcus xanthus* [79], and the Hat protein from *Synechocystis sp.* PCC6803, required for control of high affinity transport of inorganic carbon [80].

While we detect proteins with similarity to WD-repeat containing Nups (including in planctomycete genomes), sequence similarity is restricted to the WD-repeat regions alone; characteristic motifs that enable Nup identification (such as the SIEGR-motif in Rae1) are absent. That WD-domains are consistently identified in genomic screens of all three domains supports the view that these are extremely ancient [10], [11], [77], but WD-repeat containing nucleoporins, like other Nups, appear to be a eukaryote-specific innovation.

### Conclusions

The number of features that can be traced back to LECA is truly stunning, and includes the nucleus and endomembrane systems [67], [68], [81], [82], [83], linear chromosomes with telomeres [84], mitochondria [85], [86], peroxisomes [87], the cell division apparatus, mitosis and meiosis [88], [89], [90], [91], [92], phagocytosis [81], [93], [94], introns and the spliceosomal apparatus [95] and sterol synthesis [96]. Our screen for NPC components further establishes the view that LECA was a complex entity, and enables a complex nuclear pore to be ascribed to LECA, building on and confirming the conclusions of earlier studies [10], [11].

However, the immense gap between eukaryote Nucleoporins and the limited detection of related components in either bacterial or archaeal genomes leaves us no closer to establishing how these structures evolved. Mans et al. aptly referred to this as an ‘event horizon’ [10], [97], and we note that while the availability of additional eukaryote genomes is leading to a successively clearer picture of the nature of the LECA, screens of bacteria and archaea are not narrowing this gap. Structural screens and experimental characterisation are generating important new functional data, such as with the recent characterisation of structural proteins resembling eukaryote membrane-coat proteins in *Gemmata obscuriglobus* [98], [99]. However it is difficult to place such data within the context of eukaryote stem evolution as multiple interpretations are possible.

In the current case, the emerging picture is of an extremely well conserved set of vesicle-coating complexes across eukaryotes, with a similar conclusion possible for the NPC. As all these complexes are traceable to the eukaryote root, it is not formally possible to fully evaluate the protocoatomer hypothesis [18] using comparative genomic data. While some have advocated gene phylogenies [83], Nups show low levels of sequence conservation, complicating attempts to examine the deep phylogeny of the related components of vesicle coats and the NPC. Having said that, the predictive power of the protocoatomer hypothesis is clear: a prediction of this hypothesis is that, if the NPC dates back to LECA, then so should at least one set of vesicle-coating complex components. We can uncontroversially assign the entire set of coatomer complex components from COPI, COPII and clathrin-containing complexes to LECA.

Comparative genomic studies have the power to generate a broad overview of evolutionary conservation, and are in this respect helpful tools in understanding the evolution of cellular structures. Such studies can therefore provide a valuable starting point for focused investigation of the cell biology of a specific species. At the same time, they are dependent upon experimental observation, but can also suggest fruitful avenues for subsequent experimental study. Further investigation of the evolution of variant mitoses (broadly classified as open and closed) may well be worthwhile within the context of the evolution of the nuclear pore complex.

## Materials and Methods

Nup sequences were collected, aligned and alignments vetted as previously described [9]. As conservation between fungi and metazoan sequences was in some cases poor, separate fungal and metazoan alignments were created where necessary. Alignments were used to build local and global hmm profiles using HMMER 2.3.2 (http://hmmer.wustl.edu/) [21]. Species from which training data were derived are given in Table 2. Using hmmsearch from the HMMER package, annotated protein sequences derived from eukaryote genomes (given in Table 2) were screened for nucleoporin homologs.

Candidate Nup homologs were assessed using domain information in UniProt (http://www.uniprot.org/) and PFAM (http://pfam.sanger.ac.uk) [22], as well as our examination of all alignments. Sequences lacking typical motifs/domains associated with a given Nup were removed from the analysis. All remaining candidate Nup sequences were back-blasted (blastp) against the non-redundant database (NCBI). Candidates that returned best hits against other proteins were removed.

For any given eukaryote genome, where no homologs were detected for a particular Nup, the genome was screened using Nups from closely related species using blastp and tblastn.

Planctomycete genome sequences (*Gemmata obscuriglobus, Kuenenia stuttgartiensis, Planctomyces maris, Planctomyces limnophilus, Rhodopirellula baltica*) were additionally queried with our profile HMMs using Genomewise from the Wise 2.2.0 package [23].

Sequences for the individual components of the COPI, COPII and Clathrin coatomer complexes in *S. cerevisiae* were retrieved from the SGD database (http://www.yeastgenome.org) using the respective vesicle coat names as query. Sequences were used to seed initial PSI blast searches [24] against the nr protein database at NCBI. Sequences were evaluated by means of reciprocal blastp searches, as above. Alignments from the obtained sequences were generated using probcons [25] and profile hmms were created from alignments for local and global hmm profile searches. All profiles were calibrated to increase search sensitivity. Sequences obtained were evaluated as described above for Nups.

As an aid in assigning orthology, phylogenetic networks (NeighborNet [26]) were built for NPC and coatomer components using SplitsTree [27], [28]. Phylogenetic trees were constructed using raxML 7.2.2 [29] and BioNJ [30], [31]. Phylogenies were reliable for coatomer components but not across Nups. Full Nup alignments (in clustal format) and coatomer trees (in splitstree format) are provided as supplementary material (supplementary File S1).

## Supporting Information

Table S1 Accession numbers/gene IDs for all candidate Nups in Table 2.
(0.74 MB DOC)

Table S2 Accession/gene IDs for Coatomer complex protein homologs from Table 4.
(0.04 MB XLS)

Table S3 Hits for WD repeat proteins in archaeal and bacterial genomes.
(0.03 MB XLS)

Figure S1 Unrooted BioNJ tree Ndc1. (JTT, γ, 100 bootstrap replicates). Trichomonas vaginalis & Phytophthora infestans are highlighted in blue.
(0.37 MB DOC)

Figure S2 Neighbour-Joining tree of Apm1 and Apm2. Apm2 is restricted to the Saccharomycetes and likely evolved via gene duplication. The position of the Apm2 from Yarrowia lipolytica is poorly supported and likely spurious. The tree (BioNJ, JTT, γ, 100 BS replicates) was generated from protein sequence alignments.
(0.18 MB DOC)

Figure S3 Unrooted PhyML tree of Apl1 and Apl2. Vertebrate Apl1 (blue) and Apl2 evolved via gene duplication. Apl1 from fungi (dark blue) appear paralogous to vertebrate Apl1, and the results do not support evolution by duplication and divergence from fungal Apl2. The tree was generated from protein sequence alignments using the phylogeny.fr server (Dereeper A, et al. 2008 Nucleic Acids Res. 36:W465-9). Branch support (approximate likelihood ratio test: SH-like). Similar topologies were obtained with both ML and neighbor-joining methods, and with a range of parameters and models.
(1.00 MB DOC)

File S1 Alignments and phylogenies.
(2.39 MB ZIP)

## References

[1] Alcazar-Roman AR, Tran EJ, Guo S, Wente SR (2006). Inositol hexakisphosphate and Gle1 activate the DEAD-box protein Dbp5 for nuclear mRNA export.. Nat Cell Biol.

[2] Tran EJ, Wente SR (2006). Dynamic nuclear pore complexes: life on the edge.. Cell.

[3] Cronshaw JM, Krutchinsky AN, Zhang W, Chait BT, Matunis MJ (2002). Proteomic analysis of the mammalian nuclear pore complex.. J Cell Biol.

[4] Rout MP, Aitchison JD (2000). Pore relations: nuclear pore complexes and nucleocytoplasmic exchange.. Essays Biochem.

[5] Lim RY, Aebi U, Stoffler D (2006). From the trap to the basket: getting to the bottom of the nuclear pore complex.. Chromosoma.

[6] D'Angelo MA, Hetzer MW (2008). Structure, dynamics and function of nuclear pore complexes.. Trends Cell Biol.

[7] Suntharalingam M, Wente SR (2003). Peering through the pore: nuclear pore complex structure, assembly, and function.. Dev Cell.

[8] Beck M, Forster F, Ecke M, Plitzko JM, Melchior F (2004). Nuclear pore complex structure and dynamics revealed by cryoelectron tomography.. Science.

[9] Neumann N, Jeffares DC, Poole AM (2006). Outsourcing the Nucleus: Nuclear Pore Complex Genes are no Longer Encoded in Nucleomorph Genomes.. Evol Bioinform Online.

[10] Mans BJ, Anantharaman V, Aravind L, Koonin EV (2004). Comparative genomics, evolution and origins of the nuclear envelope and nuclear pore complex.. Cell Cycle.

[11] Bapteste E, Charlebois RL, MacLeod D, Brochier C (2005). The two tempos of nuclear pore complex evolution: highly adapting proteins in an ancient frozen structure.. Genome Biol.

[12] Miao M, Ryan KJ, Wente SR (2006). The integral membrane protein Pom34p functionally links nucleoporin subcomplexes.. Genetics.

[13] Heath IB (1980). Variant mitoses in lower eukaryotes: indicators of the evolution of mitosis?. International Review of Cytology.

[14] Ribeiro KC, Pereira-Neves A, Benchimol M (2002). The mitotic spindle and associated membranes in the closed mitosis of trichomonads.. Biol Cell.

[15] Brochier C, Philippe H (2002). Phylogeny: a non-hyperthermophilic ancestor for bacteria.. Nature.

[16] Fuerst JA (2005). Intracellular compartmentation in planctomycetes.. Annu Rev Microbiol.

[17] Martin W, Koonin EV (2006). Introns and the origin of nucleus-cytosol compartmentalization.. Nature.

[18] Devos D, Dokudovskaya S, Alber F, Williams R, Chait BT (2004). Components of coated vesicles and nuclear pore complexes share a common molecular architecture.. PLoS Biol.

[19] Fath S, Mancias JD, Bi X, Goldberg J (2007). Structure and organization of coat proteins in the COPII cage.. Cell.

[20] Hsia KC, Stavropoulos P, Blobel G, Hoelz A (2007). Architecture of a coat for the nuclear pore membrane.. Cell.

[21] Eddy SR (1998). Profile hidden Markov models.. Bioinformatics.

[22] Finn RD, Tate J, Mistry J, Coggill PC, Sammut SJ (2008). The Pfam protein families database.. Nucleic Acids Res.

[23] Birney E, Clamp M, Durbin R (2004). GeneWise and Genomewise.. Genome Res.

[24] Altschul SF, Madden TL, Schaffer AA, Zhang J, Zhang Z (1997). Gapped BLAST and PSI-BLAST: a new generation of protein database search programs.. Nucleic Acids Res.

[25] Do CB, Mahabhashyam MS, Brudno M, Batzoglou S (2005). ProbCons: Probabilistic consistency-based multiple sequence alignment.. Genome Res.

[26] Bryant D, Moulton V (2004). Neighbor-net: an agglomerative method for the construction of phylogenetic networks.. Mol Biol Evol.

[27] Huson DH (1998). SplitsTree: analyzing and visualizing evolutionary data.. Bioinformatics.

[28] Kloepper TH, Huson DH (2008). Drawing explicit phylogenetic networks and their integration into SplitsTree.. BMC Evol Biol.

[29] Stamatakis A, Hoover P, Rougemont J (2008). A rapid bootstrap algorithm for the RAxML Web servers.. Syst Biol.

[30] Gascuel O (1997). BIONJ: an improved version of the NJ algorithm based on a simple model of sequence data.. Mol Biol Evol.

[31] Dereeper A, Guignon V, Blanc G, Audic S, Buffet S (2008). Phylogeny.fr: robust phylogenetic analysis for the non-specialist.. Nucleic Acids Res.

[32] DeGrasse JA, DuBois KN, Devos D, Siegel TN, Sali A (2009). Evidence for a shared nuclear pore complex architecture that is conserved from the last common eukaryotic ancestor.. Mol Cell Proteomics.

[33] Dill KA, Ozkan SB, Weikl TR, Chodera JD, Voelz VA (2007). The protein folding problem: when will it be solved?. Curr Opin Struct Biol.

[34] Ritchie DW (2008). Recent progress and future directions in protein-protein docking.. Curr Protein Pept Sci.

[35] Lane CE, van den Heuvel K, Kozera C, Curtis BA, Parsons BJ (2007). Nucleomorph genome of Hemiselmis andersenii reveals complete intron loss and compaction as a driver of protein structure and function.. Proc Natl Acad Sci U S A.

[36] Lane CE, Archibald JM (2006). Novel nucleomorph genome architecture in the cryptomonad genus hemiselmis.. J Eukaryot Microbiol.

[37] Morrison HG, McArthur AG, Gillin FD, Aley SB, Adam RD (2007). Genomic minimalism in the early diverging intestinal parasite Giardia lamblia.. Science.

[38] Stechmann A, Cavalier-Smith T (2003). The root of the eukaryote tree pinpointed.. Curr Biol.

[39] Stechmann A, Cavalier-Smith T (2002). Rooting the eukaryote tree by using a derived gene fusion.. Science.

[40] Liu HL, De Souza CP, Osmani AH, Osmani SA (2009). The three fungal transmembrane nuclear pore complex proteins of aspergillus nidulans are dispensable in the presence of an intact An-Nup84-120 complex.. Mol Biol Cell.

[41] Minge MA, Silberman JD, Orr RJ, Cavalier-Smith T, Shalchian-Tabrizi K (2009). Evolutionary position of breviate amoebae and the primary eukaryote divergence.. Proc Biol Sci.

[42] Roger AJ, Simpson AG (2009). Evolution: revisiting the root of the eukaryote tree.. Curr Biol.

[43] Benchimol M (2004). Giardia lamblia: behavior of the nuclear envelope.. Parasitol Res.

[44] Hampl V, Hug L, Leigh JW, Dacks JB, Lang BF (2009). Phylogenomic analyses support the monophyly of Excavata and resolve relationships among eukaryotic “supergroups”.. Proc Natl Acad Sci U S A.

[45] Osmani AH, Davies J, Liu HL, Nile A, Osmani SA (2006). Systematic deletion and mitotic localization of the nuclear pore complex proteins of Aspergillus nidulans.. Mol Biol Cell.

[46] Stavru F, Hulsmann BB, Spang A, Hartmann E, Cordes VC (2006). NDC1: a crucial membrane-integral nucleoporin of metazoan nuclear pore complexes.. J Cell Biol.

[47] Mansfeld J, Guttinger S, Hawryluk-Gara LA, Pante N, Mall M (2006). The conserved transmembrane nucleoporin NDC1 is required for nuclear pore complex assembly in vertebrate cells.. Mol Cell.

[48] Rout MP, Aitchison JD, Suprapto A, Hjertaas K, Zhao Y (2000). The yeast nuclear pore complex: composition, architecture, and transport mechanism.. J Cell Biol.

[49] Hawryluk-Gara LA, Shibuya EK, Wozniak RW (2005). Vertebrate Nup53 interacts with the nuclear lamina and is required for the assembly of a Nup93-containing complex.. Mol Biol Cell.

[50] Hawryluk-Gara LA, Platani M, Santarella R, Wozniak RW, Mattaj IW (2008). Nup53 is required for nuclear envelope and nuclear pore complex assembly.. Mol Biol Cell.

[51] Marelli M, Lusk CP, Chan H, Aitchison JD, Wozniak RW (2001). A link between the synthesis of nucleoporins and the biogenesis of the nuclear envelope.. J Cell Biol.

[52] Sazer S (2005). Nuclear envelope: nuclear pore complexity.. Curr Biol.

[53] De Souza CP, Osmani SA (2007). Mitosis, not just open or closed.. Eukaryot Cell.

[54] De Souza CP, Osmani AH, Hashmi SB, Osmani SA (2004). Partial nuclear pore complex disassembly during closed mitosis in Aspergillus nidulans.. Curr Biol.

[55] King N, Westbrook MJ, Young SL, Kuo A, Abedin M (2008). The genome of the choanoflagellate Monosiga brevicollis and the origin of metazoans.. Nature.

[56] Karpov SA, Mylnikov AP (1993). Preliminary observations on the ultrastructure of mitosis in choanoflagellates.. European Journal of Protistology.

[57] Antonin W, Franz C, Haselmann U, Antony C, Mattaj IW (2005). The integral membrane nucleoporin pom121 functionally links nuclear pore complex assembly and nuclear envelope formation.. Mol Cell.

[58] Theisen U, Straube A, Steinberg G (2008). Dynamic rearrangement of nucleoporins during fungal “open” mitosis.. Mol Biol Cell.

[59] Mochizuki T, Tanaka S, Watanabe S (1987). Ultrastructure of the mitotic apparatus in Cryptococcus neoformans.. J Med Vet Mycol.

[60] Fitzpatrick DA, Logue ME, Stajich JE, Butler G (2006). A fungal phylogeny based on 42 complete genomes derived from supertree and combined gene analysis.. BMC Evol Biol.

[61] Keeling PJ, Fast NM (2002). Microsporidia: biology and evolution of highly reduced intracellular parasites.. Annu Rev Microbiol.

[62] Katinka MD, Duprat S, Cornillot E, Metenier G, Thomarat F (2001). Genome sequence and gene compaction of the eukaryote parasite Encephalitozoon cuniculi.. Nature.

[63] Taylor FJR (1999). Ultrastructure as a Control for Protistan Molecular Phylogeny American naturalist 1 5 4.

[64] Devos D, Dokudovskaya S, Williams R, Alber F, Eswar N (2006). Simple fold composition and modular architecture of the nuclear pore complex.. Proc Natl Acad Sci U S A.

[65] Dacks JB, Field MC, Hirt RP, Horner DS (2004). Eukaryotic Cell Evolution from a Comparative Genomic Perspective: The Endomembrane System.. Organelles, Genomes and Eukaryote Phylogeny: An Evolutionary Synthesis in the Age of Genomics.

[66] Field MC, Gabernet-Castello C, Dacks JB, Jekely G (2006). Reconstructing the evolution of the endocytic system: insights from genomics and molecular cell biology.. Evolution of the Eukaryotic Endomembrane System and Cytoskeleton.

[67] Dacks JB, Peden AA, Field MC (2009). Evolution of specificity in the eukaryotic endomembrane system.. Int J Biochem Cell Biol.

[68] Dacks JB, Field MC (2007). Evolution of the eukaryotic membrane-trafficking system: origin, tempo and mode.. J Cell Sci.

[69] Schledzewski K, Brinkmann H, Mendel RR (1999). Phylogenetic analysis of components of the eukaryotic vesicle transport system reveals a common origin of adaptor protein complexes 1, 2, and 3 and the F subcomplex of the coatomer COPI.. J Mol Evol.

[70] Dacks JB, Poon PP, Field MC (2008). Phylogeny of endocytic components yields insight into the process of nonendosymbiotic organelle evolution.. Proc Natl Acad Sci U S A.

[71] Samson RY, Obita T, Freund SM, Williams RL, Bell SD (2008). A role for the ESCRT system in cell division in archaea.. Science.

[72] Stepp JD, Pellicena-Palle A, Hamilton S, Kirchhausen T, Lemmon SK (1995). A late Golgi sorting function for Saccharomyces cerevisiae Apm1p, but not for Apm2p, a second yeast clathrin AP medium chain-related protein.. Mol Biol Cell.

[73] Cowles CR, Odorizzi G, Payne GS, Emr SD (1997). The AP-3 adaptor complex is essential for cargo-selective transport to the yeast vacuole.. Cell.

[74] Panek HR, Stepp JD, Engle HM, Marks KM, Tan PK (1997). Suppressors of YCK-encoded yeast casein kinase 1 deficiency define the four subunits of a novel clathrin AP-like complex.. EMBO J.

[75] Yeung BG, Phan HL, Payne GS (1999). Adaptor complex-independent clathrin function in yeast.. Mol Biol Cell.

[76] Roberg KJ, Crotwell M, Espenshade P, Gimeno R, Kaiser CA (1999). LST1 is a SEC24 homologue used for selective export of the plasma membrane ATPase from the endoplasmic reticulum.. J Cell Biol.

[77] Smith TF, Gaitatzes C, Saxena K, Neer EJ (1999). The WD repeat: a common architecture for diverse functions.. Trends in Biochemical Sciences.

[78] Li D, Roberts R (2001). WD-repeat proteins: structure characteristics, biological function, and their involvement in human diseases.. Cell Mol Life Sci.

[79] White DJ, Hartzell PL (2000). AglU, a protein required for gliding motility and spore maturation of Myxococcus xanthus, is related to WD-repeat proteins.. Mol Microbiol.

[80] Hisbergues M, Gaitatzes CG, Joset F, Bedu S, Smith TF (2001). A noncanonical WD-repeat protein from the cyanobacterium Synechocystis PCC6803: structural and functional study.. Protein Sci.

[81] Jékely G (2003). Small GTPases and the evolution of the eukaryotic cell.. Bioessays.

[82] Jékely G (2007). Origin of eukaryotic endomembranes: a critical evaluation of different model scenarios.. Adv Exp Med Biol.

[83] Field MC, Dacks JB (2009). First and last ancestors: reconstructing evolution of the endomembrane system with ESCRTs, vesicle coat proteins, and nuclear pore complexes.. Curr Opin Cell Biol.

[84] Nakamura TM, Cech TR (1998). Reversing time: origin of telomerase.. Cell.

[85] van der Giezen M, Tovar J (2005). Degenerate mitochondria.. EMBO Rep.

[86] Esser C, Ahmadinejad N, Wiegand C, Rotte C, Sebastiani F (2004). A genome phylogeny for mitochondria among alpha-proteobacteria and a predominantly eubacterial ancestry of yeast nuclear genes.. Mol Biol Evol.

[87] Gabaldon T, Snel B, van Zimmeren F, Hemrika W, Tabak H (2006). Origin and evolution of the peroxisomal proteome.. Biol Direct.

[88] Eme L, Moreira D, Talla E, Brochier-Armanet C (2009). A complex cell division machinery was present in the last common ancestor of eukaryotes.. PLoS One.

[89] Dacks J, Roger AJ (1999). The first sexual lineage and the relevance of facultative sex.. J Mol Evol.

[90] Ramesh MA, Malik SB, Logsdon JM (2005). A phylogenomic inventory of meiotic genes; evidence for sex in Giardia and an early eukaryotic origin of meiosis.. Curr Biol.

[91] Cavalier-Smith T (2002). Origins of the machinery of recombination and sex.. Heredity.

[92] Egel R, Penny D, Lankenau DH, Egel R (2008). On the origin of meiosis in eukaryotic evolution: Coevolution of meiosis and mitosis from feeble beginnings;.

[93] Cavalier-Smith T (2002). The phagotrophic origin of eukaryotes and phylogenetic classification of Protozoa.. Int J Syst Evol Microbiol.

[94] Jékely G (2008). Origin of the nucleus and Ran-dependent transport to safeguard ribosome biogenesis in a chimeric cell.. Biol Direct.

[95] Collins L, Penny D (2005). Complex spliceosomal organization ancestral to extant eukaryotes.. Mol Biol Evol.

[96] Desmond E, Gribaldo S (2009). Phylogenomics of sterol synthesis: insights into the origin, evolution and diversity of a key eukaryotic feature.. Genome Biol Evol.

[97] Jékely G (2005). Glimpsing over the event horizon: evolution of nuclear pores and envelope.. Cell Cycle.

[98] Santarella-Mellwig R, Franke J, Jaedicke A, Gorjanacz M, Bauer U (2010). The compartmentalized bacteria of the planctomycetes-verrucomicrobia-chlamydiae superphylum have membrane coat-like proteins.. PLoS Biol.

[99] Lonhienne TG, Sagulenko E, Webb RI, Lee KC, Franke J (2010). Endocytosis-like protein uptake in the bacterium Gemmata obscuriglobus.. Proc Natl Acad Sci U S A.

[100] Collins LJ, Poole AM, Penny D (2003). Using ancestral sequences to uncover potential gene homologues.. Appl Bioinformatics.

