# Supplementary material for: Comparative Genomic Evidence for a Complete Nuclear Pore Complex in the Last Eukaryotic Common Ancestor
Source: PLoS One. 2010 Oct 8;5(10):e13241. doi: 10.1371/journal.pone.0013241 (PMC2951903; doi:10.1371/journal.pone.0013241)
Supplement: Table S1 — Accession numbers/gene IDs for all candidate Nups in Table 2. (0.74 MB DOC) [file pone.0013241.s001.doc]

**Table S1: Accession numbers/gene IDs for all candidate Nups in Table 2**

| *Species* | Group | TPR_MLP1 |
| --- | --- | --- |
| *Aspergillus nidulans* | Fungi | gi:67538658 |
| *Cryptococcus neoformans* | Fungi | gi:134118642 |
| *Pichia stipitis* | Fungi | 0 |
| *Ustilago maydis* | Fungi | gi:71005112 |
| *Schizosaccharomyces pombe* | Fungi | ACC:SPCC162.08c |
| *Ciona intestinalis* | Opisthokonts, | ACC:estExt_fgenesh3_pg.C_chr_14p0106 |
| *Encephalitozoon cuniculi* | Opisthokonts | 0 |
| *Monosiga brevicollis* | Opisthokonts | ACC:estExt_fgenesh1_pg.C_50184 |
| *Ostreococcus tauri* | Plantae | gi:116057299 |
| *Populus trichocarpa* | Plantae | ACC:estExt_fgenesh4_pg.C_LG_VIII0693 |
| *Volvox carterii* | Plantae | ACC:fgenesh4_pg.C_scaffold_12000164 |
| *Physcomitrella patens* | Plantae | gi:168001236 |
| *Chlamydomonas*  *reinhardii* | Plantae | 0 |
| *Theileria parva* | Chromalveolates  Apicomplexa | 0 |
| *Tetrahymena thermophila* | Chromalveolates | 0 |
| *Toxoplasma gondii* | Chromalveolates | 0 |
| *Plasmodium berghei* | Chromalveolata | 0 |
| *Phytophthora infestans* | Chromalveolates | ACC:PITT_07789 |
| *Phaeodactylum tricornutum* | Chromalveolates | ACC: fgenesh1_pg.C_chr_8000078 |
| *Thalassiosira pseudonana* | Chromalveolates | Acc: 106561 |
| *Entamoeba histolytica* | Amoebozoa | 0 |
| *Dictyostelium discoideum* | Amoebozoa | ACC:DDB0187764 |
| *Leishmania major* | Excavates | (ACC:LinJ35_V3.0200) |
| *Naegleria gruberi* | Excavates | ACC:fgeneshNG_pg.scaffold_30000037 |
| *Trichomonas vaginalis* | Excavates | 0- |
| *Giardia lamblia* | Diplomonad | 0 |

| *Species* | Group | Nup93 |
| --- | --- | --- |
| *Aspergillus nidulans* | Fungi | AN6980.2  (gi:67541633) |
| *Cryptococcus neoformans* | Fungi | cn03290  (gi:58265062) |
| *Pichia stipitis* | Fungi | 76301 |
| *Ustilago maydis* | Fungi | UM03813.1 |
| *Schizosaccharomyces pombe* | Fungi | SPCC1620.11 |
| *Ciona intestinalis* | Opisthokonts,  seasquirt | 213542  (gi:198434694) |
| *Encephalitozoon cuniculi* | Opisthokonts | gi: 85014213 |
| *Monosiga brevicollis* | Plantae | 32309 |
| *Ostreococcus tauri* | Plantae | 0 |
| *Populus trichocarpa* | Plantae | 823177 |
| *Volvox carterii* | Plantae | 120776 |
| *Physcomitrella patens* | Plantae | 159641 |
| *Chlamydomonas* | Plantae | fgenesh2_pg.C_scaffold_96000002 |
| *Theileria parva* | Chromalveolates | 0 |
| *Tetrahymena thermophila* | Chromalveolates | gi:118378662 |
| *Toxoplasma gondii* | Chromalveolates | 0 |
| *Plasmodium berghei* | Chromalveolates | 0 |
| *Phytophthora infestans* | Chromalveolates | PITG_00262 |
| *Phaeodactylum tricornutum* | Chromalveolates | 47393 |
| *Thalassiosira pseudonana* | Chromalveolates | Acc: 119439 |
| *Entamoeba histolytica* | Amoebozoa | 0 |
| *Dictyostelium discoideum* | Amoebozoa | DDB0235249 |
| *Leishmania major* | Excavates | LinJ36_V3.2640 |
| *Naegleria gruberi* | Excavates | estExt_fgeneshHS_pg.C_60121 |
| *Trichomonas vaginalis* | Excavates | 89808.m00121  gi:123449498 |
| *Trypanosoma brucei* | Excavates | Tb10.6k15.3670 |

| *Species* | Group | Nup153 |
| --- | --- | --- |
| *Aspergillus nidulans* | Fungi | 0 |
| *Cryptococcus neoformans* | Fungi | ACC:cn01634  Gi:58259149 |
| *Pichia stipitis* | Fungi | ACC:82669  gi:150864554 |
| *Ustilago maydis* | Fungi | ACC:UM05489.1 |
| *Schizosaccharomyces pombe* | Fungi | ACC:SPAC30D11.04c |
| *Ciona intestinalis* | Opisthokonts, | ACC:294569  gi:118344210 |
| *Encephalitozoon cuniculi* | Opisthokonts | 0 |
| *Monosiga brevicollis* |  |  |
| *Ostreococcus tauri* | Plantae | ACC:24368  gi:145345344 |
| *Populus trichocarpa* | Plantae | ACC:770750 |
| *Volvox carterii* | Plantae | 0 |
| *Physcomitrella patens* | Plantae | 0 |
| *Theileria parva* | Chromalveolates | 0 |
| *Tetrahymena thermophila* | Chromalveolates | 0 |
| *Toxoplasma gondii* | Chromalveolates | 0 |
| *Plasmodium berghei* | Chromalveolates | 0 |
| *Phytophthora infestans* | Chromalveolates | PITT_01591 |
| *Phaeodactylum tricornutum* | Chromalveolates | 43752 |
| *Thalassiosira pseudonana* | Diatom | Acc: 122784 |
| *Entamoeba histolytica* | Amoebozoa | 0 |
| *Dictyostelium discoideum* | Amoebozoa | gi|66818619 |
| *Leishmania major* | Excavates | 0 |
| *Naegleria gruberi* | Excavates | estExt_fgeneshHS_pg.C_60106 |
| *Trichomonas vaginalis* | Excavates |  |

| *Species* | Group | Gle2 |
| --- | --- | --- |
| *Aspergillus nidulans* | Fungi | gi:67521844 |
| *Cryptococcus neoformans* | Fungi | gi:58261972 |
| *Pichia stipitis* | Fungi | gi: 150865022 |
| *Ustilago maydis* | Fungi | GI:71019357 |
| *Schizosaccharomyces pombe* | Fungi | 19113576 |
| *Ciona intestinalis* | Opisthokonts,  seasquirt | ACC:236689 |
| *Encephalitozoon cuniculi* | Opisthokonts | gi|19074406 |
| *Monosiga brevicollis* | Opisthokonts | gi:167526688 |
| *Ostreococcus tauri* | Plantae | GI:145347967 |
| *Populus trichocarpa* | Plantae | ACC:estExt_fgenesh4_pg.C_LG_III1507 |
| *Volvox carterii* | Plantae | ACC:estExt_Genewise1Plus.C_10307 |
| *Physcomitrella patens* | Plantae | ACC:estExt_fgenesh1_pg.C_330083  gi:168011685 |
| *Chlamydomonas reinhardtii* | Plantae | ACC:estExt_gwp_1W.C_380004 |
| *Theileria parva* | Chromalveolates | ACC:525.m06229  GI:71032051 |
| *Tetrahymena thermophila* | Chromalveolates | GI:118353302 |
| *Toxoplasma gondii* | Chromalveolates | ACC:59.m03621 |
| *Plasmodium berghei* | Chromalveolates | 0 |
| *Phytophthora infestans* | Chromalveolates | ACC:PITT_13431 |
| *Phaeo* | Chromalveolates | ACC:gw1.1.450.1 |
| *Thalassiosira pseudonana* | Chromalveolates | Acc: 109699 |
| *Entamoeba histolytica* | Amoebozoa | gi:67477229 |
| *Dictyostelium discoideum* | Amoebozoa | GI:66810355 |
| *Leishmania major* | Excavates | gi:146074949 |
| *Naegleria gruberi* | Excavates | ACC:estExt_fgeneshHS_kg.C_370001 |
| *Trichomonas vaginalis* | Excavates | 0 |
| *Giardia lamblia lamblia* | Diplomonad  Excavates | ACC:GL50803_102890 |
| *Trypanosoma brucei* | Excavates | Tb09_160_2360 |

| *Species* | Group | Seh1 |
| --- | --- | --- |
| *Aspergillus nidulans* | Fungi | gi:67539438 |
| *Cryptococcus neoformans* | Fungi | gi:58262718 |
| *Pichia stipitis* | Fungi | gi:150865606 |
| *Ustilago maydis* | Fungi |  |
| *Schizosaccharomyces pombe* | Fungi | ACC:SPAC15F9  gi:19114663 |
| *Ciona intestinalis* | Opisthokonts, | gi:198429345 |
| *Encephalitozoon cuniculi* | Opisthokonts |  |
| *Monosiga brevicollis* | Opisthokonts | gi:167520840 |
| *Ostreococcus tauri* | Plantae | gi:145350434 |
| *Populus trichocarpa* | Plantae | gi:118483516 |
| *Volvox carterii carterii* | Plantae | ACC:estExt_fgenesh4_kg.C_700007 |
| *Physcomitrella patens* | Plantae | gi:168050975 |
| *Chlamydo* |  | ACC: fgenesh2_pg.C_scaffold_19000005 |
| *Theileria parva* | Chromalveolates | 0 |
| *Tetrahymena thermophila* | Chromalveolates | gi:146171706 |
| *Toxoplasma gondii* | Chromalveolates | 0 |
| *Plasmodium berghei* | Chromalveolata | 0 |
| *Phytophthora infestans* |  | ACC:PITT_20611 |
| *Phaeodactylum tricornutum* | Diatom (Stramenopiles) | ACC:fgenesh1_pg.C_chr_5000122 |
| *Entamoeba histolytica* | Amoebozoa | 0 |
| *Dictyostelium discoideum* | Amoebozoa | ACC:DDB0169145  gi:66818211 |
| *Leishmania major* | Excavates | gi:146096231 |
| *Trypanosoma brucei* | Excavates | Tb11.01.5410 |
| *Naegleria gruberi* | Excavates | ACC:fgeneshHS_pg.scaffold_85000021 |
| *Trichomonas vaginalis* | Excavates |  |

| *Species* | Group | Sec13 |
| --- | --- | --- |
| *Aspergillus nidulans* | Fungi | ACC:AN4317.2  gi:67528222 |
| *Cryptococcus neoformans* | Fungi | gi:58270942 |
| *Pichia stipitis* | Fungi | gi:150863818 |
| *Ustilago maydis* | Fungi | ACC:UM02245.1  gi:71010445 |
| *Schizosaccharomyces pombe* | Fungi | ACC:SPBC215.15  gi:3873552 |
| *Ciona intestinalis* | Opisthokonts,  seasquirt | gi:198416254 |
| *Encephalitozoon cuniculi* | Opisthokonts | gi|19074945 |
| *Monosiga brevicollis* | Opisthokonts | gi:167515858 |
| *Ostreococcus tauri* | Plantae | gi:145352438 |
| *Populus trichocarpa* | Plantae | gi:118481620 |
| *Volvox carterii carterii* | Plantae | ACC:estExt_Genewise1Plus.C_120064 |
| *Physcomitrella patens* | Plantae | gi:168031228 |
| *Chlamydomonas reinhardtii* | Plantae | ACC:Chlre2_kg.scaffold_54000021 |
| *Theileria parva* | Chromalveolates | 0 |
| *Tetrahymena thermophila* | Chromalveolates | gi:146169504 |
| *Toxoplasma gondii* | Chromalveolates | ACC:20.m03668 |
| *Plasmodium berghei* | Chromalveolates | ACC:PB001149.01.0  gi:68073671 |
| *Phytophthora infestans* | Chromalveolates | ACC:PITG_12694 |
| *Phaeodactylum tricornutum* | Chromalveolates | ACC: phatr1_ua_pm.chr_1000110 |
| *Thalassiosira pseudonana* | Chromalveolates | Acc:123710 |
| *Cryptosporididium hominis* | Chromalveolates | Chro.80472 |
| *Entamoeba histolytica* | Amoebozoa | gi:67467554 |
| *Dictyostelium discoideum* | Amoebozoa | gi:66801741 |
| *Leishmania major* | Excavates | gi:146096042  gi:146091717 |
| *Naegleria gruberi* | Excavates | ACC:estExt_fgeneshHS_pg.C_100040 |
| *Trichomonas vaginalis* | Excavates | gi:154420049 |
| *Giardia lamblia lamblia* |  | ACC:GL50803_137698  Gi:159116672 |
| *Trypanosoma brucei* | Excavates | sec13_Tb10.61.2630  Tb11.01.0420 |

| *Species* | Group | Pom121 |
| --- | --- | --- |
| *Aspergillus nidulans* | Fungi | 0 |
| *Cryptococcus neoformans* | Fungi | 0 |
| *Pichia stipitis* | Fungi | 0 |
| *Ustilago maydis* | Fungi | 0 |
| *Schizosaccharomyces pombe* | Fungi | 0 |
| *Ciona intestinalis* | Opisthokonts | 0 |
| *Encephalitozoon cuniculi* | Opisthokonts | 0 |
| *Monosiga brevicollis* | Opisthokonts | 0 |
| *Ostreococcus tauri* | Plantae | 0 |
| *Populus trichocarpa* | Plantae | 0 |
| *Volvox carterii carterii* | Plantae | 0 |
| *Physcomitrella patens* | Plantae | 0 |
| *Theileria parva* | Chromalveolates | 0 |
| *Tetrahymena thermophila* | Chromalveolates | 0 |
| *Toxoplasma gondii* | Chromalveolates | 0 |
| *Plasmodium berghei* | Chromalveolates | 0 |
| *Phytophthora infestans* | Chromalveolates | 0 |
| *Phaeodactylum tricornutum* | Chromalveolates | 0 |
| *Entamoeba histolytica* | Amoebozoa | 0 |
| *Dictyostelium discoideum* | Amoebozoa | 0 |
| *Leishmania major* | Excavates | 0 |
| *Naegleria gruberi* | Excavates | 0 |
| *Trichomonas vaginalis* | Excavates | 0 |

| *Species* | Group | GP210 |
| --- | --- | --- |
| *Aspergillus nidulans* | Fungi | 0 |
| *Cryptococcus neoformans* | Fungi | 0 |
| *Pichia stipitis* | Fungi | 0 |
| *Ustilago maydis* | Fungi | 0 |
| *Schizosaccharomyces pombe* | Fungi | 0 |
| *Ciona intestinalis* | Opisthokonts,  seasquirt | gi:198414653 |
| *Encephalitozoon cuniculi* | Opisthokonts | 0 |
| *Monosiga brevicollis* | Opisthokonts | gi:167526609 |
| *Ostreococcus tauri* | Plantae | 0 |
| *Populus trichocarpa* | Plantae | ACC:eugene3.00011990 |
| *Volvox carterii carterii* | Plantae | 0 |
| *Physcomitrella patens* | Plantae | gi:168019688 |
| *Theileria parva* | Chromalveolates | ACC: 530.m02994  gi:71027085 |
| *Tetrahymena thermophila* | Chromalveolates | Gi:118357834 |
| *Toxoplasma gondii* | Chromalveolates | ACC:113.m00794 |
| *Plasmodium berghei* | Chromalveolates | 0 |
| *Phytophthora infestans* | Chromalveolates | 0 |
| *Phaeodactylum tricornutum* | Chromalveolates | 0 |
| *Cryptosporidium* | Chromalveolates | Gi: 67602359 |
| *Entamoeba histolytica* | Amoebozoa | gi:67477774 |
| *Dictyostelium discoideum* | Amoebozoa | gi:66805893 |
| *Leishmania major* | Excavates | 0 |
| *Naegleria gruberi* | Excavates | ACC:HS.pg.C_280005 |
| *Trichomonas vaginalis* | Excavates | ACC:81997.m00113  gi:121902208 |

| *Species* | Group | POM34 |
| --- | --- | --- |
| *Aspergillus nidulans* | Fungi | Seq. with NPCC domain in other Aspergillus |
| *Cryptococcus neoformans* | Fungi |  |
| *Pichia stipitis* | Fungi | ACC:fgenesh1_pg.C_chr_4.1000125 |
| *Ustilago maydis* | Fungi | 0 |
| *Schizosaccharomyces pombe* | Fungi | ACC:SPAC1002.02 |
| *Ciona intestinalis* | Opisthokonts,  seasquirt | 0 |
| *Encephalitozoon cuniculi* | Opisthokonts | 0 |
| *Monosiga brevicollis* | Opisthokonts | 0 |
| *Ostreococcus tauri* | Plantae | 0 |
| *Populus trichocarpa* | Plantae | 0 |
| *Volvox carterii carterii* | Plantae | 0 |
| *Physcomitrella patens* | Plantae | 0 |
| *Theileria parva* | Chromalveolates | 0 |
| *Tetrahymena thermophila* | Chromalveolates | 0 |
| *Toxoplasma gondii* | Chromalveolates | 0 |
| *Plasmodium berghei* | Chromalveolates | 0 |
| *Phytophthora infestans* | Chromalveolates | 0 |
| *Phaeodactylum tricornutum* | Chromalveolates | 0 |
| *Entamoeba histolytica* | Amoebozoa | 0 |
| *Dictyostelium discoideum* | Amoebozoa | 0 |
| *Leishmania major* | Excavates | 0 |
| *Naegleria gruberi* | Excavates | 0 |
| *Trichomonas vaginalis* | Excavates | 0 |

| *Species* | Group | POM152 |
| --- | --- | --- |
| *Aspergillus nidulans* | Fungi | ACC:AN3454.2 |
| *Cryptococcus neoformans* | Fungi | ACC:cn03050 |
| *Laccaria bicolor* | Fungi | 170085593 |
| *Ustilago maydis* | Fungi | ACC:UM03963.1 |
| *Coprinossis cinea* | Fungi | 169844937 |
| *Malassesezia* | Fungi | gi|164663187 |
| *Phanerochaete chrysosporium* | Fungi | fgenesh1_pg.C_scaffold_13000286 |
| *Schizosaccharomyces pombe* | Fungi | ACC:SPBC29A10.07 |
| *Pichia stipitis* | Fungi | ACC:e_gwh1.2.1.58.1 |
| *Rhizopus oryzae* | Fungi | ACC: RO3G_04411.3 |
| *Ciona intestinalis* | Opisthokonts, | 0 |
| *Encephalitozoon cuniculi* | Opisthokonts | 0 |
| *Monosiga brevicollis* | Plantae | 0 |
| *Ostreococcus tauri* | Plantae | 0 |
| *Populus trichocarpa* | Plantae | 0 |
| *Volvox carterii carterii* | Plantae | 0 |
| *Physcomitrella patens* | Plantae | 0 |
| *Theileria parva* | Chromalveolates | 0 |
| *Tetrahymena thermophila* | Chromalveolates | 0 |
| *Toxoplasma gondii* | Chromalveolates | 0 |
| *Plasmodium berghei* | Chromalveolates | 0 |
| *Phytophthora infestans* | Chromalveolates | 0 |
| *Phaeodactylum tricornutum* | Chromalveolates | 0 |
| *Entamoeba histolytica* | Amoebozoa | 0 |
| *Dictyostelium discoideum* | Amoebozoa | 0 |
| *Leishmania major* | Excavates | 0 |
| *Naegleria gruberi* | Excavates | 0 |
| *Trichomonas vaginalis* | Excavates | 0 |

| *Species* | Group | Nup50 |
| --- | --- | --- |
| *Aspergillus nidulans* | Fungi | ACC:AN5485.2 |
| *Cryptococcus neoformans* | Fungi | ACC:cn01191 |
| *Pichia stipitis* | Fungi | ACC:estExt_gwp_genewisePlus_worm.C_chr_5.10008  gi:150865775 |
| *Ustilago maydis* | Fungi | ACC:UM03950.1 |
| *Schizosaccharomyces pombe* | Fungi | ACC:SPCC18B5.07c |
| *Ciona intestinalis* | Opisthokonts,  seasquirt | gi:198418022 |
| *Encephalitozoon cuniculi* | Opisthokonts | 0 |
| *Monosiga brevicollis* | Opisthokonts | gi:167525072 |
| *Ostreococcus tauri* | Plantae | gi:145351040 |
| *Populus trichocarpa* | Plantae | ACC:fgenesh4_pg.C_LG_I000992 |
| *Volvox carterii carterii* | Plantae |  |
| *Physcomitrella patens* | Plantae | 0 |
| *Theileria parva* | Chromalveolates | 0 |
| *Tetrahymena thermophila* | Chromalveolates | 0 |
| *Toxoplasma gondii* | Chromalveolates | 0 |
| *Plasmodium berghei* | Chromalveolates | 0 |
| *Thalassiossira pseudonana* | Chromalveolates | Gi: 223993929 |
| *Phytophthora infestans* | Chromalveolates | 0 |
| *Phaeodactylum tricornutum* | Diatom (Stramenopiles) | 48647 |
| *Entamoeba histolytica* | Amoebozoa | 0 |
| *Dictyostelium discoideum* | Amoebozoa | ACC:DDB0205337 |
| *Leishmania major* | Excavates | 0 |
| *Naegleria gruberi* | Excavates | fgeneshHS_pg.scaffold_80000035 |
| *Trichomonas vaginalis* | Excavates | 0 |

| *Species* | Group | Gle1 |
| --- | --- | --- |
| *Aspergillus nidulans* | Fungi | gi:67517973 |
| *Cryptococcus neoformans* | Fungi | GI:58259877 |
| *Pichia stipitis* | Fungi | GI:126138970 |
| *Ustilago maydis* | Fungi | gi:71005568 |
| *Schizosaccharomyces pombe* | Fungi | gi:19111893 |
| *Ciona intestinalis* | Opisthokonts, | ACC:estExt_fgenesh3_pg.C_440068 |
| *Encephalitozoon cuniculi* | Opisthokonts |  |
| *Monosiga brevicollis* | Opisthokonts | Gi:167536461 |
| *Ostreococcus tauri* | Plantae | gi:145353472 |
| *Populus trichocarpa* | Plantae | ACC: grail3.0009032601 |
| *Volvox carterii carterii* | Plantae | 0 |
| *Physcomitrella patens* | Plantae | gi:168059956 |
| *Chlamydomonas reinhardtii* | Plantae | ACC:Chlre2_kg.scaffold_12000184  gi:159466156 |
| *Theileria parva* | Chromalveolates | 0 |
| *Tetrahymena thermophila* | Chromalveolates | 0 |
| *Toxoplasma gondii* | Chromalveolates | 0 |
| *Plasmodium berghei* | Chromalveolates | 0 |
| *Phytophthora infestans* | Chromalveolates | PITG_16006 |
| *Phaeodactylum tricornutum* | Chromalveolates | ACC:estExt_fgenesh1_pg.C_chr_10532 |
| *Thalassiosira pseudonana* | Chromalveolates | Gi: 220969892 |
| *Cryptosporidium hominis* | Chromalveolates | Chro.70044 |
| *Entamoeba histolytica* | Amoebozoa | 0 |
| *Dictyostelium discoideum* | Amoebozoa | ACC:DDB0189646  GI:66828123 |
| *Leishmania major* | Excavates | 0 |
| *Naegleria gruberi* | Excavates | ACC:fgeneshHS_pg.scaffold_2000183 |
| *Trichomonas vaginalis* | Excavates | 0 |

| *Species* | Group | Nup155 |
| --- | --- | --- |
| *Aspergillus nidulans* | Fungi | ACC:AN6738.2 |
| *Cryptococcus neoformans* | Fungi | ACC:CNBD5020 |
| *Pichia stipitis* | Fungi | ACC:estExt_fgenesh1_pg.C_chr_1.10380 |
| *Ustilago maydis* | Fungi | ACC:UM03853.1 |
| *Schizosaccharomyces pombe* | Fungi | ACC:SPAC890.06 |
| *Ciona intestinalis* | Opisthokonts,  seasquirt | gi:198422013 |
| *Encephalitozoon cuniculi* | Opisthokonts | gi:19074297 |
| *Monosiga brevicollis* | Opisthokonts | ACC:fgenesh2_pg.scaffold_2000632 |
| *Ostreococcus tauri* | Plantae | ACC:fgenesh1_pg.C_Chr_10000160 |
| *Populus trichocarpa* | Plantae | 0 |
| *Volvox carterii carterii* | Plantae | ACC: fgenesh5_synt.49__25 |
| *Physcomitrella patens* | Plantae | 0 |
| *Chlamydomonas reinhardtii* | Plantae | gi:159472697 |
| *Theileria parva* | Chromalveolates | 0 |
| *Tetrahymena thermophila* | Chromalveolates | ACC:125.m00095 |
| *Toxoplasma gondii* | Chromalveolates | 0 |
| *Plasmodium berghei* | Chromalveolates | 0 |
| *Phytophthora infestans* | Chromalveolates | ACC:PITT_07103 |
| *Phaeodactylum tricornutum* | Chromalveolates | ACC: fgenesh1_pg.C_chr_7000232 |
| *Thalassiosira pseudonana* | Chromalveolates | Acc: 162409 |
| *Cryptosporidium hominis* | Chromalveolates | gi:67587446 |
| *Entamoeba histolytica* | Amoebozoa | 0 |
| *Dictyostelium discoideum* | Amoebozoa | ACC:DDB0235243 |
| *Leishmania major* | Excavates | ACC:LinJ36_V3.7220 |
| *Naegleria gruberi* | Excavates | ACC:fgeneshNG_pg.scaffold_1000134 |
| *Trichomonas vaginalis* | Excavates | gi:123488164  gi:123456555 |
| *Giardia lamblia lamblia* | Excavates | GL50803_112408  Gi:159118577 |
| *Ttrypanosoma brucei* | Excavates | Tb10.6k15.2350 |

| *Species* | Group | Nup35 |
| --- | --- | --- |
| *Aspergillus nidulans* | Fungi |  |
| *Cryptococcus neoformans* | Fungi | ACC:cn09027 |
| *Pichia stipitis* | Fungi | ACC: e_gww1.3.1.606.1 |
| *Ustilago maydis* | Fungi | 0 |
| *Schizosaccharomyces pombe* | Fungi | ACC:SPAC19E9.01c |
| *Ciona intestinalis* | Opisthokonts, | ACC:estExt_fgenesh3_pg.C_640091 |
| *Encephalitozoon cuniculi* | Opisthokonts | gi:19074443 |
| *Monosiga brevicollis* | Opisthokonts | ACC:estExt_fgenesh2_pg.C_290079 |
| *Ostreococcus tauri* | Plantae | gi:145344257 |
| *Populus trichocarpa* | Plantae | ACC: eugene3.00011347 |
| *Volvox carterii carterii* | Plantae | ACC:e_gw1.13.92.1 |
| *Physcomitrella patens* | Plantae | gi:168067203 |
| *Chlamydomonas reinhardtii*  *reinhardtii* | Plantae | ACC: Chlre2_kg.scaffold_20000237 |
| *Theileria parva* | Chromalveolates | 0 |
| *Tetrahymena thermophila* | Chromalveolates | 0 |
| *Toxoplasma gondii* | Chromalveolates | 0 |
| *Plasmodium berghei* | Chromalveolates | 0 |
| *Phytophthora infestans* | Chromalveolates | ACC:PITT_12683 |
| *Phaeodactylum tricornutum* | Chromalveolates | ACC:estExt_fgenesh1_pg.C_chr_110318 |
| *Thalassiosira pseudonana* | Chromalveolates | Gi: 224000121 |
| *Entamoeba histolytica* | Amoebozoa | 0 |
| *Dictyostelium discoideum* | Amoebozoa | ACC:DDB0169513 |
| *Leishmania major* | Excavates | 0 |
| *Naegleria gruberi* | Excavates | 0 |
| *Trichomonas vaginalis* | Excavates | gi:123410202 |
| *Giardia lamblia lamblia* | Excavates | ACC:GL50803_3598  gi: 159112342 |

| *Species* | Group | Nup54 |
| --- | --- | --- |
| *Aspergillus nidulans* | Fungi | ACC:AN1064  gi:67517765. |
| *Cryptococcus neoformans* | Fungi | ACC:cn10246  gi:58260146 |
| *Pichia stipitis* | Fungi | gi:150864724 |
| *Ustilago maydis* | Fungi | ACC:UM02319.1  gi:71011513 |
| *Schizosaccharomyces pombe* | Fungi | gi:19112773 |
| *Ciona intestinalis* | Opisthokonts,  seasquirt | ACC: gw1.02q.1361.1 |
| *Encephalitozoon cuniculi* | Opisthokonts | 0 |
| *Monosiga brevicollis* | Opisthokonts | ACC: estExt_fgenesh2_pg.C_160028 |
| *Ostreococcus tauri* | Plantae | gi:145341740 |
| *Populus trichocarpa* | Plantae | ACC:fgenesh4_pm.C_LG_VIII000750 |
| *Volvox carterii carterii* | Plantae | ACC: fgenesh5_synt.54__45 |
| *Physcomitrella patens* | Plantae | gi: 168012954  gi:168039195 |
| *Chlamydomonas reinhardtii* | Plantae | ACC: fgenesh2_pg.C_scaffold_20000065 |
| *Theileria parva* | Chromalveolates | 0 |
| *Tetrahymena thermophila* | Chromalveolates | ACC: 23.m00229  gi:146162229 |
| *Toxoplasma gondii* | Chromalveolates | ACC:50.m03239  541.m01245 |
| *Plasmodium berghei* | Chromalveolates | 0 |
| *Phytophthora infestans* | Chromalveolates | ACC:PITG_14603 |
| *Phaeodactylum tricornutum* | Chromalveolates | ACC:estExt_fgenesh1_pg.C_chr_20605 |
| *Thalassiosira pseudonana* | Diatom | Acc: 113018 |
| *Entamoeba histolytica* | Amoebozoa | 0 |
| *Dictyostelium discoideum* | Amoebozoa | ACC:DDB0235246 |
| *Leishmania major* | Excavates | gi: 146095571 |
| *Naegleria gruberi* | Excavates | ACC:fgeneshNG_pg.scaffold_70000015 |
| *Trichomonas vaginalis* | Excavates | ACC:90434.m00219 |
| *Giardi lamblia* | Excavate | GL50803_7240 |
| *Trypanosoma brucei* | Excavate | Tb927_4_5200 |

| *Species* | Group | Nup58 |
| --- | --- | --- |
| *Aspergillus nidulans* | Fungi | ACC:AN2431.2  gi:67523951 |
| *Cryptococcus neoformans* | Fungi | gi:134106739 |
| *Pichia stipitis* | Fungi | gi:126276026 |
| *Ustilago maydis* | Fungi | UM01418.1  gi:71005798 |
| *Schizosaccharomyces pombe* | Fungi | SPAC22G7.09c  gi:19113970 |
| *Ciona intestinalis* | Opisthokonts,  seasquirt | Gi:198433114 |
| *Encephalitozoon cuniculi* | Opisthokonts | 0 |
| *Monosiga brevicollis* |  | estExt_fgenesh2_pg.C_70011 gi:167520947 |
| *Ostreococcus tauri* | Plantae | 0 |
| *Populus trichocarpa* | Plantae | ACC: gw1.VII.3169.1 |
| *Volvox carterii* | Plantae | ACC:estExt_fgenesh4_pg.C_290123 |
| *Physcomitrella patens* | Plantae | gi:168025155 |
| *Chlamydomonas reinhardtii* | Plantae | fgenesh2_pg.C_scaffold_45000088  gi: 159482286 |
| *Theileria parva* | Chromalveolates | 0 |
| *Tetrahymena thermophila* | Chromalveolates | 0 |
| *Toxoplasma gondii* | Chromalveolates | 0 |
| *Plasmodium berghei* | Chromalveolates | 0 |
| *Phytophthora infestans* | Chromalveolates | ACC:PITT_05012 |
| *Phaeodactylum tricornutum* | Chromalveolates | 0 |
| *Entamoeba histolytica* | Amoebozoa | 0 |
| *Dictyostelium discoideum* | Amoebozoa | gi:66815839 |
| *Leishmania major* | Excavates | Gi:146092981 |
| *Naegleria gruberi* | Excavates | ACC:fgeneshHS_pg.scaffold_3000230 |
| *Trichomonas vaginalis* | Excavates | 0 |

| *Species* | Group | Nup62 |
| --- | --- | --- |
| *Aspergillus nidulans* | Fungi | gi:67534149 |
| *Cryptococcus neoformans* | Fungi | gi:58266044 |
| *Pichia stipitis* | Fungi | gi:150863747 |
| *Ustilago maydis* | Fungi | gi:71020851 |
| *Schizosaccharomyces pombe* | Fungi | ACC:SPAC26A3.15c  gi:19115070 |
| *Ciona intestinalis* | Opisthokonts,  seasquirt | gi:198428491 |
| *Encephalitozoon cuniculi* | Opisthokonts | gi:19074982 |
| *Monosiga brevicollis* | Opisthokonts | gi:167519865 |
| *Ostreococcus tauri* | Plantae | gi:119358789 (blast) |
| *Populus trichocarpa* | Plantae | ACC:fgenesh4_pg.C_scaffold_40000043 |
| *Volvox carterii carterii* | Plantae | ACC: estExt_fgenesh4_pg.C_320012 |
| *Physcomitrella patens* | Plantae | gi:168032308 |
| *Chlamydomonas reinhardtii* | Plantae | Chlre2_kg.scaffold_28000156 |
| *Theileria parva* | Chromalveolates | 0 |
| *Tetrahymena thermophila* | Chromalveolates | 0 |
| *Toxoplasma gondii* | Chromalveolates | ACC:59.m03706 |
| *Plasmodium berghei* | Chromalveolata | 0 |
| *Phytophthora infestans* | Chromalveolates | ACC:PITT_10416 |
| *Phaeodactylum tricornutum* | Chromalveolates | ACC:estExt_fgenesh1_pg.C_chr_290096 |
| *Thalassiosira pseudonana* | Chromalveolates | Acc: 105779 |
| *C. hominis* | Chromalveolates | Chro.20379 gi:67592622: |
| *Entamoeba histolytica* | Amoebozoa | ACC:51.m00158 |
| *Dictyostelium discoideum* | Amoebozoa | gi:66820658 |
| *Leishmania major* | Excavates | gi:146096784 |
| *Naegleria gruberi* | Excavates | ACC:estExt_fgeneshHS_pg.C_460045 |
| *Trichomonas vaginalis* | Excavates | 87974.m00049  gi:123412152 |
| *Trypanosoma brucei* | Excavates | Tb11_01_7200 |

| *Species* | Group | Nup120 |
| --- | --- | --- |
| *Aspergillus nidulans* | Fungi | gi:67521558 |
| *Cryptococcus neoformans* | Fungi | gi:58266180 |
| *Pichia stipitis* | Fungi | ACC:estExt_gwp_genewisePlus_worm.C_chr_1.11565 |
| *Ustilago maydis* | Fungi | gi:71004240 |
| *Schizosaccharomyces pombe* | Fungi | ACC:SPBC3B9.16c  gi:19113466 |
| *Ciona intestinalis* | Opisthokonts,  seasquirt | ACC:estExt_fgenesh3_pg.C_chr_12p0290 |
| *Encephalitozoon cuniculi* | Opisthokonts | 0 |
| *Monosiga brevicollis* | Opisthokonts | gi:167536226 |
| *Ostreococcus tauri* | Plantae | gi:145354226 |
| *Populus trichocarpa* | Plantae | ACC: gw1.I.8199.1 |
| *Volvox carterii carterii* | Plantae | ACC:fgenesh4_pg.C_scaffold_48000049 |
| *Physcomitrella patens* | Plantae | ACC:estExt_fgenesh1_pg.C_3320022  gi:168061027 |
| *Theileria parva* | Chromalveolates | 0 |
| *Tetrahymena thermophila* | Chromalveolates | 0 |
| *Toxoplasma gondii* | Chromalveolates | 0 |
| *Plasmodium berghei* | Chromalveolata |  |
| *Phytophthora infestans* |  | ACC:PITT_18244 |
| *Phaeodactylum tricornutum* | Diatom | 0 |
| *Entamoeba histolytica* | Amoebozoa | 0 |
| *Dictyostelium discoideum* | Amoebozoa | gi:166240053 |
| *Leishmania major* | Excavates | 0 |
| *Naegleria gruberi* | Excavates | ACC:fgeneshNG_pg.scaffold_1000224 |
| *Trichomonas vaginalis* | Excavates | 0 |

| *Species* | Group | NDC1 |
| --- | --- | --- |
| *Aspergillus nidulans* | Fungi | ACC:AN4417.2  gi:67528438 |
| *Cryptococcus neoformans* | Fungi | gi134111176 |
| *Pichia stipitis* | Fungi | gi:150865459 |
| *Ustilago maydis* | Fungi | UM06416.1  gi:71024667 |
| *Coprinopsis cinerea* | Fungi | Gi: 169857185 |
| *Malassezia globosa* | Fungi | Gi: 164655325 |
| *Phanerochaete chrysosporium* | Fungi | fgenesh1_pg.C_scaffold_10000288 |
| *Laccaria bicolor* | Fungi | Gi: 170107891 |
| *Schizosaccharomyces pombe* | Fungi | gi: 63054586 |
| *Rhizopus oryzae* | Fungi | RO3G_09895.3 |
| *Ciona intestinalis* | Opisthokonts, | Gi:198423796 |
| *Encephalitozoon cuniculi* | Opisthokonts | 0 |
| *Monosiga brevicollis* | Opisthokonts | 0 |
| *Ostreococcus tauri* | Plantae | ost_03_011_101  gi:145344267 |
| *Populus trichocarpa* | Plantae | fgenesh4_pg.C_LG_I000121 |
| *Volvox carterii* | Plantae | Tblastn  scaffold_57 (912469 bp) : 422202:425849 (3648 bp) |
| *Physcomitrella patens* | Plantae | e_gw1.405.2.1  gi:168064987 |
| *Arabidopsis thaliana* | Plantae | gi:18410306 |
| *Chlamydomonas reinhardtii* | Plantae | Gi:159470299 |
| *Oryza sativa* | Plantae | gi:125540702 |
| *Theileria parva* | Chromalveolates | 0 |
| *Tetrahymena thermophila* | Chromalveolates | 0 |
| *Toxoplasma gondii* | Chromalveolates | 0 |
| *Plasmodium berghei* | Chromalveolata | 0 |
| *Phytophthora infestans* | Chromalveolates | PITG_03144 |
| *Phaeodactylum tricornutum* | Chromalveolates | 0 |
| *Entamoeba histolytica* | Amoebozoa | 0 |
| *Dictyostelium discoideum* | Amoebozoa | 0 |
| *Leishmania major* | Excavates | 0 |
| *Naegleria gruberi* | Excavates | 0 |
| *Trichomonas vaginalis* | Excavates | 90867.m00167 |

| *Species* | Group | Nup214 |
| --- | --- | --- |
| *Aspergillus nidulans* | Fungi | ACC:AN2086.2 |
| *Cryptococcus neoformans* | Fungi | gi:134112630 |
| *Pichia stipitis* | Fungi | ACC:estExt_gwp_genewisePlus_worm.C_chr_1.11503 |
| *Ustilago maydis* | Fungi | gi:71005140 |
| *Schizosaccharomyces pombe* | Fungi | gi:19115455 |
| *Ciona intestinalis* | Opisthokonts, | gi:198416620 |
| *Encephalitozoon cuniculi* | Opisthokonts | 0 |
| *Monosiga brevicollis* | Opisthokonts | gi:167527053 |
| *Ostreococcus tauri* | Plantae | gi:145346525 |
| *Populus trichocarpa* | Plantae | Gi:222844870 |
| *Volvox carterii carterii* | Plantae | 0 |
| *Physcomitrella patens* | Plantae | 0 |
| *Theileria parva* | Chromalveolates | 0 |
| *Tetrahymena thermophila* | Chromalveolates | 0 |
| *Toxoplasma gondii* | Chromalveolates | ACC:20.m05982 |
| *Plasmodium berghei* | Chromalveolates | 0 |
| *Phytophthora infestans* | Chromalveolates | ACC:PITT_01259 |
| *Phaeodactylum tricornutum* | Chromalveolates | 0 |
| *Entamoeba histolytica* | Amoebozoa | 0 |
| *Dictyostelium discoideum* | Amoebozoa | DDB0235251  gi:66804667 |
| *Leishmania major* | Excavates | ACC:LinJ31_V3.1080 |
| *Naegleria gruberi* | Excavates | ACC:estExt_fgeneshHS_pg.C_70079 |
| *Trichomonas vaginalis* | Excavates | 0 |

| *Species* | Group | Nup98 |
| --- | --- | --- |
| *Aspergillus nidulans* | Fungi | ACC:AN5627.2  gi:67538914 |
| *Cryptococcus neoformans* | Fungi | gi:134108552  gi:58262756 (nup98) |
| *Pichia stipitis* | Fungi | gi:150863970 (Nup98)  gi:150951070 (precursor) |
| *Ustilago maydis* | Fungi | ACC:UM05158.1  gi:71022149 |
| *Schizosaccharomyces pombe* | Fungi | gi:19115005 |
| *Ciona intestinalis* | Opisthokonts, | ACC:fgenesh3_pg.C_scaffold_260000003 |
| *Encephalitozoon cuniculi* | Opisthokonts | gi: 19073967 |
| *Monosiga brevicollis* |  | gi:167537610 |
| *Ostreococcus tauri* | Plantae | gi:145340910 |
| *Populus trichocarpa* | Plantae | ACC:fgenesh4_pg.C_LG_XI001276  ACC: eugene3.00031493 |
| *Volvox carterii* | Plantae | ACC:gw1.37.39.1 |
| *Physcomitrella patens* | Plantae | gi:168049501  gi:168049555 |
| *Chlamydomonas reinhardtii* | Plantae | ACC:Chlre2_kg.scaffold_7000270  ACC:fgenesh2_pg.C_scaffold_7000292 |
| *Theileria parva* | Chromalveolates | gi:71030568  gi:71029456  gi:71028452 |
| *Thalassiosira pseudonana pseudonanna* |  | ACC:114004  Gi_224002855 |
| *Tetrahymena thermophila* | Chromalveolates | gi:146161552  ACC:27.m00216  gi:118400911  4.m00696 |
| *Toxoplasma gondii* | Chromalveolates | ACC:55.m04836 |
| *Plasmodium berghei* | Chromalveolata | Gi:68077075 |
| *Phytophthora infestans* |  | ACC:PITG_11743 |
| *Phaeodactylum tricornutum* | Diatom (Stramenopiles) | ACC:estExt_fgenesh1_pg.C_chr_80188 |
| *Cryptosporidium hominis* |  | Chro.30276  Gi: 67581106 |
| *Entamoeba histolytica* | Amoebozoa | gi:183234251 |
| *Dictyostelium discoideum* | Amoebozoa | ACC:DDB0235244  gi:66802620 |
| *Leishmania major* | Excavates | gi:146090681 |
| *Naegleria gruberi* | Excavates | ACC:estExt_fgeneshNG_pg.C_120135 |
| *Trichomonas vaginalis* | Excavates | ACC:92921.m00297 |
| *Giardia lamblia lamblia* | Excavates | GL50803_8038 |
| *Trypanosoma brucei* | Excavates | Tb11.03.0140 |

| *Species* | Group | Nup192 |
| --- | --- | --- |
| *Aspergillus nidulans* | Fungi | gi:67515511 |
| *Cryptococcus neoformans* | Fungi | gi:134111509 |
| *Pichia stipitis* | Fungi | gi:150864613 |
| *Ustilago maydis* | Fungi | gi:71013846 |
| *Schizosaccharomyces pombe* | Fungi | ACC:SPCC290.03c |
| *Ciona intestinalis* | Opisthokonts, | gi:198421954 |
| *Encephalitozoon cuniculi* | Opisthokonts | 0 |
| *Monosiga brevicollis* | Opisthokonts | gi:167534999 |
| *Ostreococcus tauri* | Plantae | gi:145347617 |
| *Populus trichocarpa* | Plantae | ACC: grail3.0028025201 |
| *Volvox carterii* | Plantae | ACC: fgenesh5_synt.13__80 |
| *Physcomitrella patens* | Plantae | gi:168047323 |
| *Chlamydomonas reinhardtii* | Plantae | fgenesh2_pg.C_scaffold_2000307  gi:159471289 |
| *Theileria parva* | Chromalveolates | 0 |
| *Tetrahymena thermophila* | Chromalveolates | 0 |
| *Toxoplasma gondii* | Chromalveolates | 0 |
| *Plasmodium berghei* | Chromalveolates | 0 |
| *Phytophthora infestans* | Chromalveolates | ACC:PITT_06257 |
| *Phaeodactylum tricornutum* | Chromalveolates | 0 |
| *Entamoeba histolytica* | Amoebozoa | 0 |
| *Dictyostelium discoideum* | Amoebozoa | 0 |
| *Leishmania major* | Excavates | Gi: 157875703 |
| *Trypanosoma brucei* | Excavates | Gi: 72388028 |
| *Naegleria gruberi* | Excavates | ACC:estExt_fgeneshHS_pg.C_590013 |
| *Trichomonas vaginalis* | Excavates | gi:123455687 |

| *Species* | Group | Nup37 |
| --- | --- | --- |
| *Aspergillus nidulans* | Fungi | AN2773.3  gi: 67524631 |
| *Cryptococcus neoformans* | Fungi | 0 |
| *Pichia stipitis* | Fungi | 0 |
| *Ustilago maydis* | Fungi | 0 |
| *Schizosaccharomyces pombe* | Fungi | gi:19115673 |
| *Ciona intestinalis* | Opisthokonts, | gi:198430055 |
| *Encephalitozoon cuniculi* | Opisthokonts | 0 |
| *Monosiga brevicollis* |  | 0 |
| *Ostreococcus tauri* | Plantae | 0 |
| *Populus trichocarpa* | Plantae | 0 |
| *Volvox carterii* | Plantae | 0 |
| *Physcomitrella patens* | Plantae | 0 |
| *Theileria parva* | Chromalveolates | 0 |
| *Tetrahymena thermophila* | Chromalveolates | 0 |
| *Toxoplasma gondii* | Chromalveolates | 0 |
| *Plasmodium berghei* | Chromalveolates | 0 |
| *Phytophthora infestans* | Chromalveolates | 0 |
| *Phaeodactylum tricornutum* | Chromalveolates | 0 |
| *Entamoeba histolytica* | Amoebozoa | 0 |
| *Dictyostelium discoideum* | Amoebozoa | 0 |
| *Leishmania major* | Excavates | 0 |
| *Naegleria gruberi* | Excavates | 0 |
| *Trichomonas vaginalis* | Excavates | 0 |

| *Species* | Group | Nup43 |
| --- | --- | --- |
| *Aspergillus nidulans* | Fungi | 0 |
| *Cryptococcus neoformans* | Fungi | 0 |
| *Pichia stipitis* | Fungi | 0 |
| *Ustilago maydis* | Fungi | 0 |
| *Schizosaccharomyces pombe* | Fungi | 0 |
| *Ciona intestinalis* | Opisthokonts, | ACC: estExt_fgenesh3_pg.C_chr_12q0502  gi:198425061 |
| *Encephalitozoon cuniculi* | Opisthokonts | 0 |
| *Monosiga brevicollis* | Opisthokonts | gi:167517557  estExt_fgenesh1_pg.C_30391 |
| *Ostreococcus tauri* | Plantae | 0 |
| *Populus trichocarpa* | Plantae | ACC: fgenesh4_pm.C_LG_VI000554 |
| *Volvox carterii* | Plantae | 0 |
| *Physcomitrella patens* | Plantae | gi:168034421 |
| *Theileria parva* | Chromalveolates | 0 |
| *Tetrahymena thermophila* | Chromalveolates | 0 |
| *Toxoplasma gondii* | Chromalveolates | 0 |
| *Plasmodium berghei* | Chromalveolates | 0 |
| *Phytophthora infestans* | Chromalveolates | ACC:PITT_16121 |
| *Phaeodactylum tricornutum* | Chromalveolates | 0 |
| *Entamoeba histolytica* | Amoebozoa | 0 |
| *Dictyostelium discoideum* | Amoebozoa | gi:66816027 |
| *Leishmania major* | Excavates | 0 |
| *Naegleria gruberi* | Excavates | 0 |
| *Trichomonas vaginalis* | Excavates | 0 |

| *Species* | Group | Nup88 |
| --- | --- | --- |
| *Aspergillus nidulans* | Fungi | AN6143.2  gi:67539946 |
| *Cryptococcus neoformans* | Fungi | gi:58267050 |
| *Pichia stipitis* | Fungi | gi:150864788 |
| *Ustilago maydis* | Fungi | gi:71003628 |
| *Schizosaccharomyces pombe* | Fungi | gi:19113207 |
| *Ciona intestinalis* | Opisthokonts, | gi:198419446 |
| *Encephalitozoon cuniculi* | Opisthokonts | 0 |
| *Monosiga brevicollis* | Opisthokonts | 0 |
| *Ostreococcus tauri* | Plantae | gi:145346822 |
| *Populus trichocarpa* | Plantae | ACC:eugene3.00080608 |
| *Volvox carterii* | Plantae | ACC: fgenesh4_pg.C_scaffold_1000744 |
| *Physcomitrella patens* | Plantae | gi:168019291 |
| *Chlamydomonas reinhardtii* | Plantae | ACC: estExt_fgenesh2_pg.C_260018 |
| *Theileria parva* | Chromalveolates | 0 |
| *Tetrahymena thermophila* | Chromalveolates | 0 |
| *Toxoplasma gondii* | Chromalveolates | 0 |
| *Plasmodium berghei* | Chromalveolates | 0 |
| *Phytophthora infestans* | Chromalveolates | ACC:PITT_04553 |
| *Phaeodactylum tricornutum* | Chromalveolates | 0 |
| *Entamoeba histolytica* | Amoebozoa | 0 |
| *Dictyostelium discoideum* | Amoebozoa | gi:66815831 |
| *Leishmania major* | Excavates | 0 |
| *Naegleria gruberi* | Excavates | 0 |
| *Trichomonas vaginalis* | Excavates | 0 |

| *Species* | Group | Nup75/85 |
| --- | --- | --- |
| *Aspergillus nidulans* | Fungi | gi:67904244 |
| *Cryptococcus neoformans* | Fungi | ACC:cn02170 |
| *Pichia stipitis* | Fungi | ACC:fgenesh1_pm.C_chr_1.2000098 |
| *Ustilago maydis* | Fungi | ACC:UM04624.1 |
| *Schizosaccharomyces pombe* | Fungi | ACC:SPBC17G9.04c |
| *Ciona intestinalis* | Opisthokonts, | ACC:estExt_fgenesh3_pg.C_chr_03q0243 |
| *Encephalitozoon cuniculi* | Opisthokonts | 0 |
| *Monosiga brevicollis* |  | ACC:estExt_fgenesh2_pg.C_190044 |
| *Ostreococcus tauri* | Plantae | gi:145341851 |
| *Populus trichocarpa* | Plantae | ACC:estExt_fgenesh4_pg.C_LG_XVIII0252 |
| *Volvox carterii* | Plantae | ACC:fgenesh4_pg.C_scaffold_42000132 |
| *Physcomitrella patens* | Plantae | ACC: e_gw1.127.11.1 |
| *Chlamydomonas reinhardtii* | Plantae | ACC:fgenesh2_pg.C_scaffold_21000038*  gi: 159472158 |
| *Theileria parva* | Chromalveolates | 0 |
| *Tetrahymena thermophila* | Chromalveolates | 0 |
| *Toxoplasma gondii* | Chromalveolates | 0 |
| *Plasmodium berghei* | Chromalveolates | 0 |
| *Phytophthora infestans* | Chromalveolates | ACC:PITT_01257 |
| *Phaeodactylum tricornutum* | Chromalveolates | 0 |
| *Entamoeba histolytica* | Amoebozoa | 0 |
| *Dictyostelium discoideum* | Amoebozoa | ACC:DDB0235247 |
| *Leishmania major* | Excavates | 0 |
| *Naegleria gruberi* | Excavates | ACC:estExt_fgeneshHS_pg.C_410085 |
| *Trichomonas vaginalis* | Excavates | 0 |

| *Species* | Group | Nup133 |
| --- | --- | --- |
| *Aspergillus nidulans* | Fungi | AN4293.2  gi:67528174 |
| *Cryptococcus neoformans* | Fungi | gi:134117536 |
| *Pichia stipitis* | Fungi | gi:150951198 |
| *Ustilago maydis* | Fungi | gi:46098780 |
| *Schizosaccharomyces pombe* | Fungi | gi:19114827  gi:19112173 |
| *Ciona intestinalis* | Opisthokonts, | ACC:gw1.04q.663.1 |
| *Encephalitozoon cuniculi* | Opisthokonts | 0 |
| *Monosiga brevicollis* |  | 0 |
| *Ostreococcus tauri* | Plantae | ACC: ost_20_011_025 |
| *Populus trichocarpa* | Plantae | ACC:estExt_Genewise1_v1.C_LG_II1291 |
| *Volvox carterii* | Plantae | ACC:fgenesh4_pg.C_scaffold_85000034 |
| *Physcomitrella patens* | Plantae | gi:168037104 |
| *Chlamydomonas reinhardtii*  *reinhardtii* | Plantae | Gi: 159477635 (likely truncated) |
| *Theileria parva* | Chromalveolates | 0 |
| *Tetrahymena thermophila* | Chromalveolates | 0 |
| *Toxoplasma gondii* | Chromalveolates | 0 |
| *Plasmodium berghei* | Chromalveolata | 0 |
| *Phytophthora infestans* | Chromalveolates | ACC:PITT_02730 |
| *Phaeodactylum tricornutum* | Chromalveolates | Gi_ 219126421 |
| *Thalassiosira pseudonana pseudonana* | Chromalveolates | gi:220971352 |
| *Entamoeba histolytica* | Amoebozoa | 0 |
| *Dictyostelium discoideum* | Amoebozoa | ACC:DDB0235242  gi:66806945 |
| *Leishmania major* | Excavates | 0 |
| *Naegleria gruberi* | Excavates | ACC:fgeneshNG_pg.scaffold_35000161 |
| *Trichomonas vaginalis* | Excavates | 0 |

| *Species* | Group | Nup188 |
| --- | --- | --- |
| *Aspergillus nidulans* | Fungi | -gene not annotated , Osmani 2006 |
| *Cryptococcus neoformans* | Fungi | gi:134113182 |
| *Pichia stipitis* | Fungi | gi:150864400 |
| *Ustilago maydis* | Fungi | 0 |
| *Schizosaccharomyces pombe* | Fungi | gi:19114326 |
| *Ciona intestinalis* | Opisthokonts, | gi:198412885 |
| *Encephalitozoon cuniculi* | Opisthokonts | 0 |
| *Monosiga brevicollis* | Opisthokonts | 0 |
| *Ostreococcus tauri* | Plantae | gi:145346085 |
| *Populus trichocarpa* | Plantae | ACC:fgenesh4_pg.C_LG_IX000369 |
| *Volvox carterii* | Plantae | ACC:fgenesh4_pg.C_scaffold_11000114 |
| *Physcomitrella patens* | Plantae | 170225  gi:168051734 |
| *Chlamydomonas reinhardtii* | Plantae | fgenesh2_pg.C_scaffold_2000475 |
| *Theileria parva* | Chromalveolates | 0 |
| *Tetrahymena thermophila* | Chromalveolates | 0 |
| *Toxoplasma gondii* | Chromalveolates | 0 |
| *Plasmodium berghei* | Chromalveolata | 0 |
| *Phytophthora infestans* | Chromalveolates | 0 |
| *Phaeodactylum tricornutum* | Chromalveolates | 0 |
| *Entamoeba histolytica* | Amoebozoa | 0 |
| *Dictyostelium discoideum* | Amoebozoa | 0 |
| *Leishmania major* | Excavates | 0 |
| *Naegleria gruberi* | Excavates | 0 |
| *Trichomonas vaginalis* | Excavates | 0 |

| *Species* | Group | Nup107 |
| --- | --- | --- |
| *Aspergillus nidulans* | Fungi | ACC:AN1190.2 |
| *Cryptococcus neoformans* | Fungi | ACC:cn08275  gi:134115040 |
| *Pichia stipitis* | Fungi | ACC: genesh1_pg.C_chr_4.1000347 |
| *Ustilago maydis* | Fungi | ACC:UM04795.1  gi:71021423 |
| *Schizosaccharomyces pombe* | Fungi | ACC:SPBC428.01c  gi:68009955 |
| *Ciona intestinalis* | Opisthokonts, | ACC: gw1.08q.1163.1  gi:198437921 |
| *Encephalitozoon cuniculi* | Opisthokonts | 0 |
| *Monosiga brevicollis* | Opisthokonts | ACC:estExt_fgenesh2_pg.C_170028 |
| *Ostreococcus tauri* | Plantae | gi:145341857 |
| *Populus trichocarpa* | Plantae | ACC:estExt_Genewise1_v1.C_1580091 |
| *Volvox carterii* | Plantae | ACC:fgenesh4_pg.C_scaffold_55000023 |
| *Physcomitrella patens* | Plantae | gi:168033720 |
| *Chlamydomonas reinhardtii* | Plantae | Tblastn jgi  Scaffold_8: 1241663-1241980 |
| *Theileria parva* | Chromalveolates | 0 |
| *Tetrahymena thermophila* | Chromalveolates | 0 |
| *Toxoplasma gondii* | Chromalveolates | 0 |
| *Plasmodium berghei* | Chromalveolates | 0 |
| *Phytophthora infestans* | Chromalveolates | ACC:PITT_05404 |
| *Phaeodactylum tricornutum* | Chromalveolates | ACC:estExt_fgenesh1_pg.C_chr_100127 |
| *Thalassiosira pseudonana* | Chromalveolates | Acc: 114200 |
| *Entamoeba histolytica* | Amoebozoa | 0 |
| *Dictyostelium discoideum* | Amoebozoa | ACC:DDB0235236 |
| *Leishmania major* | Excavates | gI:157875121 |
| *Naegleria gruberi* | Excavates | ACC:estExt_fgeneshNG_pg.C_20291 |
| *Trichomonas vaginalis* | Excavates | gi:154421560 |
| *Trypanosoma brucei* | Excavates | gi:71754925 |
| *Giardia lamblia lamblia* | Excavates | gi: 253742276 |

| *Species* | Group | **Aladin** |
| --- | --- | --- |
| *Aspergillus nidulans* | Fungi | 0 |
| *Cryptococcus neoformans* | Fungi | 0 |
| *Pichia stipitis* | Fungi | 0 |
| *Ustilago maydis* | Fungi | 0 |
| *Schizosaccharomyces pombe* | Fungi | 0 |
| *Ciona intestinalis* | Opisthokonts, | gi:198416512 |
| *Encephalitozoon cuniculi* | Opisthokonts | 0 |
| *Monosiga brevicollis* | Opisthkonts | gi:167524507 |
| *Ostreococcus tauri* | Plantae | gi:145356562 |
| *Populus trichocarpa* | Plantae | ACC: gw1.XVI.1102.1  Gi: 118486469 |
| *Volvox carterii* | Plantae | ACC: fgenesh4_pg.C_scaffold_16000100 |
| *Physcomitrella patens* | Plantae | gi:168026846 |
| *Chlamydomonas reinhardtii* | Planta | Tblastn on jgi:  scaffold_4: 3053876-3053941 |
| *Theileria parva* | Chromalveolates | 0 |
| *Tetrahymena thermophila* | Chromalveolates | 0 |
| *Toxoplasma gondii* | Chromalveolates | 0 |
| *Plasmodium berghei* | Chromalveolates | 0 |
| *Phytophthora infestans* | Chromalveolates | ACC:PITT_03146 |
| *Phaeodactylum tricornutum* | Chromalveolates | 0 |
| *Entamoeba histolytica* | Amoebozoa | 0 |
| *Dictyostelium discoideum* | Amoebozoa | gi:66818405 |
| *Leishmania major* | Excavates | Koonin:  gi:15487196 |
| *Trypanosoma*  *brucei* | Excavates | gi:71755231 |
| *Naegleria gruberi* | Excavates | ACC:fgeneshNG_pg.scaffold_2000178 |
| *Trichomonas vaginalis* | Excavates | 0 |

| *Species* | Group | Ranbp2 |
| --- | --- | --- |
| *Aspergillus nidulans* | Fungi |  |
| *Cryptococcus neoformans* | Fungi |  |
| *Pichia stipitis* | Fungi |  |
| *Ustilago maydis* | Fungi | 0 |
| *Schizosaccharomyces pombe* | Fungi |  |
| *Ciona intestinalis* | Opisthokonts | gi: 198438399 |
| *Encephalitozoon cuniculi* | Opisthokonts | 0 |
| *Monosiga brevicollis* | Opisthokonts | estExt_fgenesh2_pg.C_30249 |
| *Ostreococcus tauri* | Plantae | 0 |
| *Populus trichocarpa* | Plantae | 0 |
| *Volvox carterii carterii* | Plantae | 0 |
| *Physcomitrella patens* | Plantae | 0 |
| *Theileria parva* | Chromalveolates | 0 |
| *Tetrahymena thermophila* | Chromalveolates | 0 |
| *Toxoplasma gondii* | Chromalveolates | 0 |
| *Plasmodium berghei* | Chromalveolates | 0 |
| *Phytophthora infestans* | Chromalveolates | 0 |
| *Phaeodactylum tricornutum* | Chromalveolates | 0 |
| *Entamoeba histolytica* | Amoebozoa | 0 |
| *Dictyostelium discoideum* | Amoebozoa | 0 |
| *Leishmania major* | Excavates | 0 |
| *Naegleria gruberi* | Excavates | 0 |
| *Trichomonas vaginalis* | Excavates | 0 |
