# Supplementary material for: Comparative Genomic Evidence for a Complete Nuclear Pore Complex in the Last Eukaryotic Common Ancestor
Source: PLoS One. 2010 Oct 8;5(10):e13241. doi: 10.1371/journal.pone.0013241 (PMC2951903; doi:10.1371/journal.pone.0013241)
Supplement: Figure S1 — Unrooted BioNJ tree Ndc1. (JTT, γ, 100 bootstrap replicates). Trichomonas vaginalis & Phytophthora infestans are highlighted in blue. (0.37 MB DOC) [file pone.0013241.s004.doc]

##
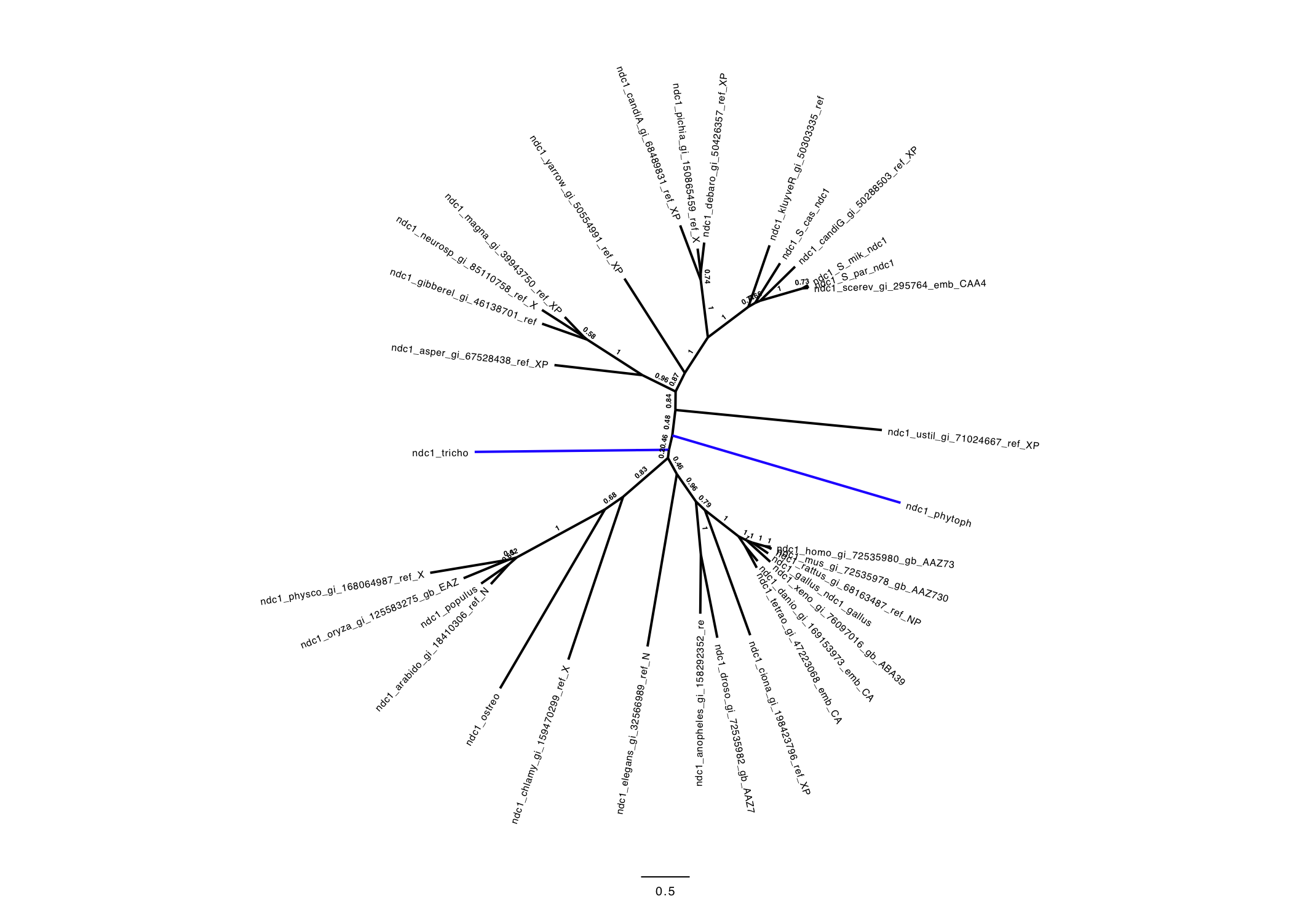


## Figure S1 – Unrooted BioNJ tree Ndc1 (JTT, γ, 100 bootstrap replicates). *Trichomonas vaginalis* & *Phytophthora infestans* are highlighted in blue.
