# Supplementary material for: Comparative Genomic Evidence for a Complete Nuclear Pore Complex in the Last Eukaryotic Common Ancestor
Source: PLoS One. 2010 Oct 8;5(10):e13241. doi: 10.1371/journal.pone.0013241 (PMC2951903; doi:10.1371/journal.pone.0013241)
Supplement: Figure S2 — Neighbour-Joining tree of Apm1 and Apm2. Apm2 is restricted to the Saccharomycetes and likely evolved via gene duplication. The position of the Apm2 from Yarrowia lipolytica is poorly supported and likely spurious. The tree (BioNJ, JTT, γ, 100 BS replicates) was generated from protein sequence alignments. (0.18 MB DOC) [file pone.0013241.s005.doc]

##
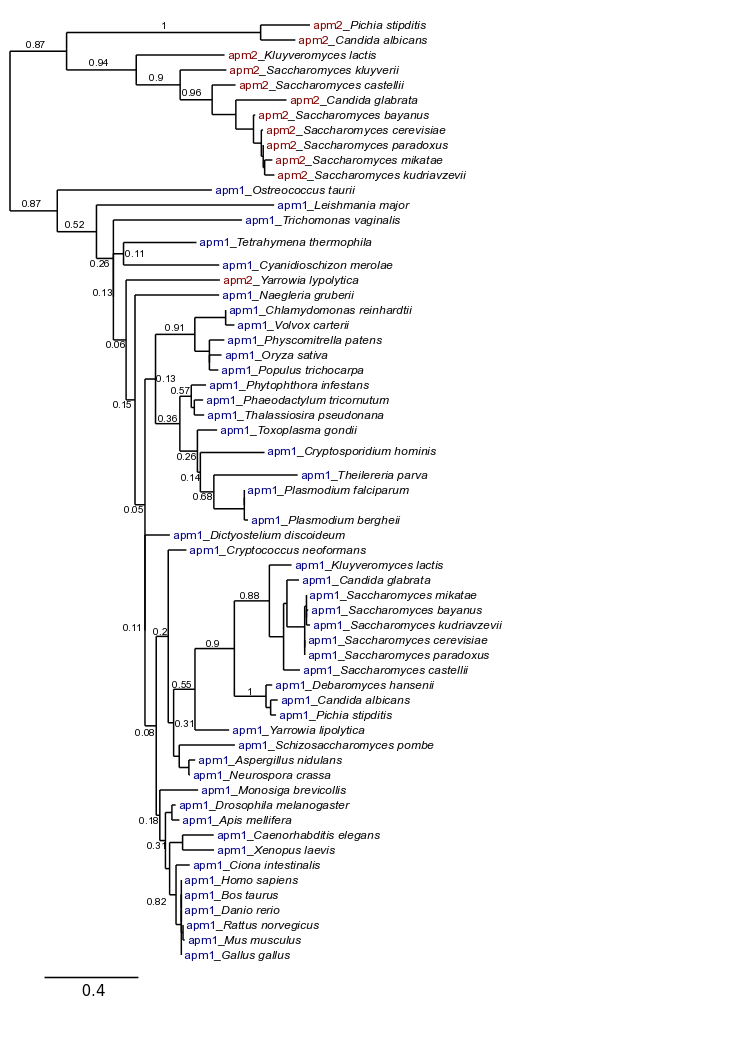


## Figure S2 – Neighbour-Joining tree of Apm1 and Apm2.

Apm2 is restricted to the Saccharomycetes and likely evolved via gene duplication. The position of the Apm2 from *Yarrowia lipolytica* is poorly supported and likely spurious. The tree (BioNJ, JTT, γ, 100 BS replicates) was generated from protein sequence alignments.
