# Supplementary material for: Comparative Genomic Evidence for a Complete Nuclear Pore Complex in the Last Eukaryotic Common Ancestor
Source: PLoS One. 2010 Oct 8;5(10):e13241. doi: 10.1371/journal.pone.0013241 (PMC2951903; doi:10.1371/journal.pone.0013241)
Supplement: Figure S3 — Unrooted PhyML tree of Apl1 and Apl2. Vertebrate Apl1 (blue) and Apl2 evolved via gene duplication. Apl1 from fungi (dark blue) appear paralogous to vertebrate Apl1, and the results do not support evolution by duplication and divergence from fungal Apl2. The tree was generated from protein sequence alignments using the phylogeny.fr server (Dereeper A, et al. 2008 Nucleic Acids Res. 36:W465-9). Branch support (approximate likelihood ratio test: SH-like). Similar topologies were obtained with both ML and neighbor-joining methods, and with a range of parameters and models. (1.00 MB DOC) [file pone.0013241.s006.doc]

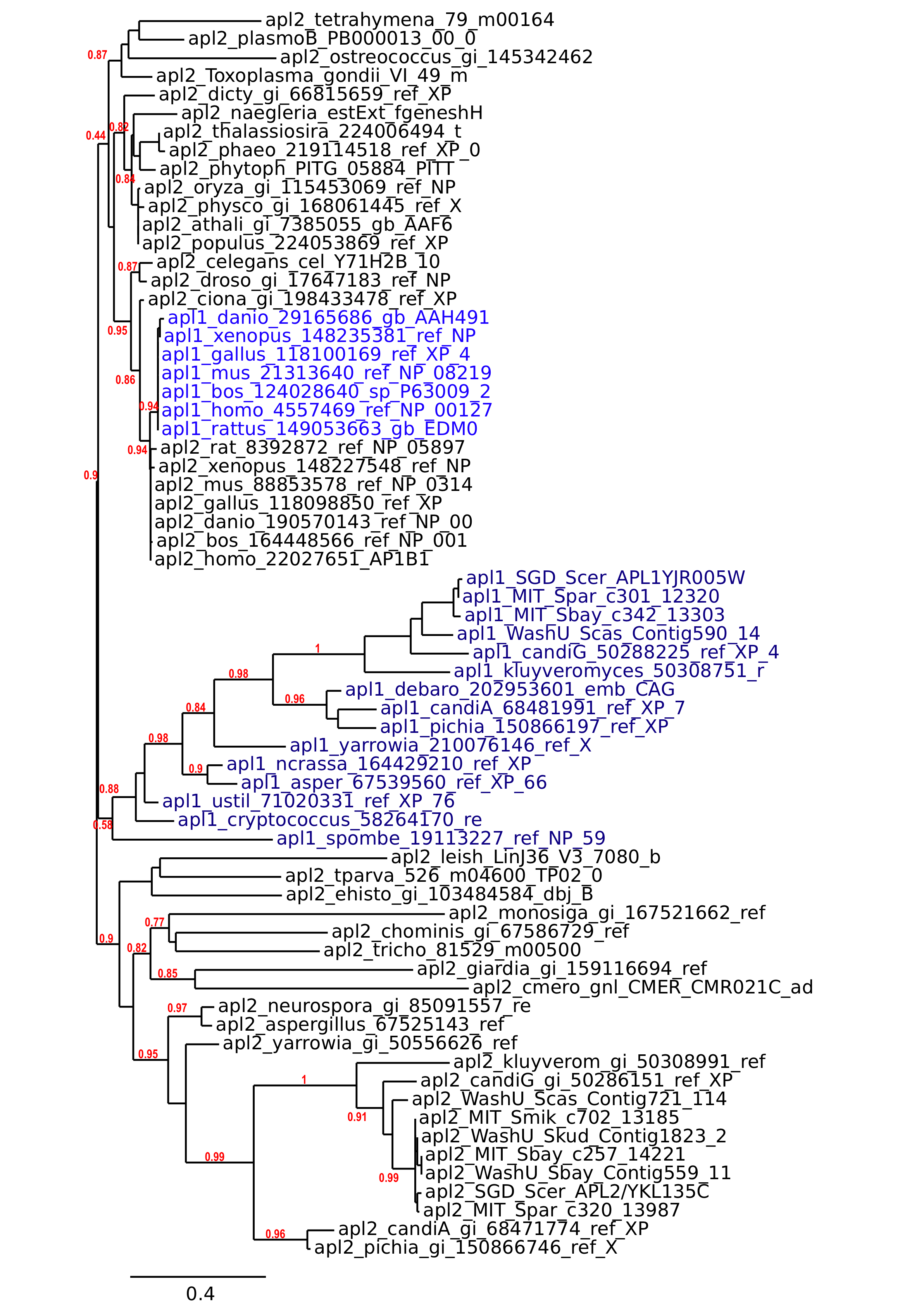


## Figure S3 – Unrooted PhyML tree of Apl1 and Apl2. Vertebrate Apl1 (blue) and Apl2 evolved via gene duplication. Apl1 from fungi (dark blue) appear paralogous to vertebrate Apl1, and the results do not support evolution by duplication and divergence from fungal Apl2. The tree was generated from protein sequence alignments using the phylogeny.fr server (Dereeper A, et al. 2008 Nucleic Acids Res. 36:W465-9). Branch support (approximate likelihood ratio test: SH-like). Similar topologies were obtained with both ML and neighbor-joining methods, and with a range of parameters and models.
