# Supplementary material for: Comparative Genomic Evidence for a Complete Nuclear Pore Complex in the Last Eukaryotic Common Ancestor
Source: PLoS One. 2010 Oct 8;5(10):e13241. doi: 10.1371/journal.pone.0013241 (PMC2951903; doi:10.1371/journal.pone.0013241)
Supplement: File S1 — Alignments and phylogenies. (2.39 MB ZIP) [file pone.0013241.s007.zip › Neumann_SI4/Information_on_alignments_trees.rtf]

Supplementary files for Neumann et al.: Comparative genomic evidence for a complete Nuclear Pore Complex in the Last Eukaryotic Common Ancestor

This folder contains clustalx alignments for all nucleoporins (see Table 2) plus splitstree files for all coatomer complex components (see Table 5). Software to read these files can be accessed from the following sites (correct, August 2010):

Clustalx
http://www.clustal.org/ 

Splitstree
http://www.splitstree.org/ 


Comments concerning length of some candidates included in alignments.
The length of some sequences (either extremely long or extremely short) may indicate a particular candidate is either incomplete or orf identification/length (by us or others) is incorrect. It is likewise possible for such sequences that the length may preclude accurate identification of sequences. These are cases where on sequence similarity we judge that all display clear sequence homology but they deviate in length from other proteins included in alignments. We stress that were we to remove all of these candidates from Table 2, this would not affect the number of Nups identified by supergroup nor would it alter reconstruction of Nup content in LECA.

Aladin
The candidate from Monosiga brevicollis is very long, but aligns well. We conclude that this sequence is most likely an Aladin homolog.

Gp210
The candidate for Cryptosporidium hominis is extremely short. However, we identified a longer C. parvum sequence, which aligns well. Both are included in the alignment, and on this basis, we conclude that Gp210 is present within the genus Cryptosporidium.

Nup54
The sequence from Volvox carteri is very long, but aligns well from position 700; Nup54 is present in all other members of Plantae screened. 
The sequence from Thalassiossira pseudonana is short, but carries conserved regions – possibly an incomplete sequence; Nup54 present in four other Chromalveolates.

Nup85
The sequence from Populus trichocarpa is very short. Given clear identification of Nup85 from other plants plus good alignment, we suspect this may be an incomplete sequence.

Nup93
The sequence from Thalassiossira pseudonana is short but aligns well with a longer sequence identified in Phaeodactylum tricornutum. A total of four Nup93 candidates were identified in Chromalveolates.

Nup120/160
The sequence from Populus trichocarpa is very short but is nearly identical to other plant sequences; Nup120 is present across all other green plants and green algae.

Nup155
Both Populus trichocarpa and Physcomitrella patens sequences are short but align well with those of other plants. Nup155 candidates are present in all other members of Plantae included in this screen.
Sequence from Thalassiossira pseudonana is also short, but aligns well across identified region; Nup155 is present in four other Chromalveolates.

Seh1
Sequences from Rattus norvegicus and Gallus gallus are both very long. Both align very well with other vertebrate sequences.

Nup35
Monosiga brevicollis candidate is very long; this may either be a fusion or a misannotation. The region showing homology to Nup35 is nevertheless very similar to other candidates; Nup35 is well conserved outside metazoa.

NSP1/Nup62
The candidate from Trichomonas vaginalis is short. The fragment contains a clear NSP1 domain; three other Nup62 candidates identified in Excavates.

Nup192/Nup205
The candidate from Aspergillus nidulans may be a fusion between a Nup205 and cullin. Nup205 is broadly conserved among the Ascomycetes.
The candidate from Naegleria gruberi is also long, but the N-terminus aligns well. The unaligned C-terminal region shows similarities to protein kinase domains, and to a hydrolase. Nup205 candidates found in four of five Excavates screened.

Nup107
The candidate from Monosiga brevicollis is large and contains a Nup107 domain in the c-terminal half. Nup107 candidates found in all 5 eukaryote supergroups.
